# Confocal microscopy-based estimation of intracellular conductivities in myocardium for modeling of the normal and infarcted heart

**DOI:** 10.1016/j.compbiomed.2022.105579

**Published:** 2022-05-03

**Authors:** Joachim Greiner, Aparna C. Sankarankutty, Thomas Seidel, Frank B. Sachse

**Affiliations:** a Institute for Experimental Cardiovascular Medicine, University Heart Center Freiburg⋅Bad Krozingen, Freiburg, Germany; b Faculty of Medicine, University of Freiburg, Freiburg, Germany; c Nora Eccles Harrison Cardiovascular Research and Training Institute, University of Utah, Salt Lake City, USA; d Department of Biomedical Engineering, University of Utah, Salt Lake City, USA; e Institute of Cellular and Molecular Physiology, Friedrich-Alexander-University of Erlangen-Nürnberg, Erlangen, Germany

**Keywords:** Cardiac modeling, Intracellular conductivities, Myocardial infarction, Confocal microscopy

## Abstract

Ventricular arrhythmias are the leading cause of mortality in patients with ischemic heart diseases, such as myocardial infarction (MI). Computational simulation of cardiac electrophysiology provides insights into these arrhythmias and their treatment. However, only sparse information is available on crucial model parameters, for instance, the anisotropic intracellular electrical conductivities. Here, we introduced an approach to estimate these conductivities in normal and MI hearts. We processed and analyzed images from confocal microscopy of left ventricular tissue of a rabbit MI model to generate 3D reconstructions. We derived tissue features including the volume fraction of myocytes $\left(V_{myo}\right)$, gap junctions-containing voxels $\left(V_{gj}\right)$, and fibrosis $\left(V_{fibrosis}\right)$. We generated models of the intracellular space and intercellular coupling. Applying numerical methods for solving Poisson’s equation for stationary electrical currents, we calculated normal $\left(\sigma_{myo,n}\right)$, longitudinal $\left(\sigma_{myo,l}\right)$, and transverse $\left(\sigma_{myo,t}\right)$ intracellular conductivities. Using linear regression analysis, we assessed relationships of conductivities to tissue features. $V_{gj}$ and $V_{myo}$ were reduced in MI vs. control, but $V_{fibrosis}$ was increased. Both $\sigma_{myo,l}$ and $\sigma_{myo,n}$ were lower in MI than in control. Differences of $\sigma_{myo,t}$ between control and MI were not significant. We found strong positive relationships of $\sigma_{myo,l}$ with $V_{myo}$ and $V_{gj}$, and a strong negative relationship with $V_{fibrosis}$. The relationships of $\sigma_{myo,n}$ with these tissue features were similar but less pronounced. Our study provides quantitative insights into the intracellular conductivities in the normal and MI heart. We suggest that our study establishes a framework for the estimation of intracellular electrical conductivities of myocardium with various pathologies.

## 1. Introduction

Ischemic heart disease is the leading cause of death worldwide [1]. Commonly, obstruction of the coronary arteries that supply blood to the myocardium underlies ischemic heart disease [2]. Complete occlusion causes myocardial infarction (MI), leading to necrosis of cardiomyocytes and scar formation. The myocardial tissue proximal to the scar, i.e. the border zone (BZ), undergoes microstructural remodeling and further functional remodeling, e.g. of myocyte electrophysiology, intercellular electrical coupling, and electrical conduction, which can result in arrhythmia. MI patients who develop arrhythmias, such as ventricular tachycardia and ventricular fibrillation, exhibit severe morbidity and high mortality risk due to sudden cardiac death [3]. Despite extensive research, however, our understanding of the complex relationships between cardiac remodeling and the development of arrhythmias following MI remains unsatisfactory.

Computer modeling of cardiac tissue electrophysiology is a promising approach to gain insights into the often complex mechanisms of arrhythmogenesis and the maintenance of arrhythmias [4,5]. Also, modeling-based planning of clinical treatment, such as ablation therapy [6,7], has been proposed.

Commonly, the computer models reflect that electrical conduction in cardiac tissues is highly anisotropic, which is caused by the 3D arrangement of myocytes and their intercellular electrical coupling through gap junction channels [8]. The conduction is affected by the geometry and orientation of the myocytes as well as the distribution and density of the gap junction channels. Connexin43 (Cx43) is the predominant protein forming gap junction channels in ventricular myocytes in the mammalian heart [9]. Remodeling of Cx43, such as lateralization and reduced expression, was reported for various cardiac pathologies and influences conduction [10,11].

Parameters of the models of electrical conduction in cardiac tissues stem from a multitude of experimental measurements, starting in the middle of the last century [4]. Parameters include the electrical tissue conductivities, volume fractions of the myocytes, extracellular space (ES), and surface-to-volume ratios of the myocytes. Similarly, experimental measurements were applied to describe electrophysiology of the myocytes by models of the sarcolemma, including different ion channels, exchangers, and pumps. Computer models of electrical conduction in diseased tissues reflect changes due to structural and functional remodeling of the tissues and cells. While cellular remodeling has been extensively characterized in experimental studies, there is a lack of information on the remodeling of the passive electrical tissue conductivities and their anisotropies [12].

Electrical conduction in cardiac tissues is commonly modeled by monodomain and bidomain models [5]. While the monodomain model considers the intracellular (myocyte) space and ES lumped as one domain, the bidomain model considers independent anisotropic conductivities for the intracellular (myocyte) space and ES. Thus, the bidomain model requires knowledge of intracellular and ES conductivities. More recently, extensions of these models incorporated additional domains for myocardial cell types other than cardiomyocytes, such as fibroblasts and myofibroblasts [12–15].

To realistically describe 3D cardiac conduction using the bidomain model, precise knowledge about the extracellular and intracellular conductivity and their anisotropies is required. In general, the conductivities were determined from experimental measurements on the heart or excised myocardium. A 3D orthogonal coordinate system was established to describe anisotropic conductivities. The three axes of the coordinate system are: 1) along the long axis of the myocytes, 2) orthogonal to the long axis of the myocytes and along the plane of interlaminar clefts, and 3) normal to the interlaminar clefts [13,14]. The conductivities can be conveniently described with tensors of second order. Our prior work developed a methodology to calculate the extracellular conductivity tensors, using computational simulations with conductivity models of cardiac tissue derived from confocal microscopy and image processing [15]. We applied this approach to conductivity calculation of normal left ventricular rabbit myocardium and various regions in the MI border zone [16]. Using statistical approaches, our work revealed fundamental relationships between tissue features and the anisotropic extracellular conductivities.

Here, we introduce a comprehensive methodology to determine the intracellular anisotropic conductivities of myocardial tissue in the normal and infarcted heart. We used high-resolution 3D images of the left ventricular (LV) myocardium obtained by scanning confocal microscopy on tissues from an established rabbit model of MI. We advanced our approaches for the segmentation of myocytes to improve accuracy and to capture in detail the intercellular coupling of myocytes via gap junctions. After image processing, we generated 3D models of intracellular space and intercellular coupling and used them to simulate electrical current flow by numerically solving Poisson’s equation. From the simulations, we derived conductivities. We found novel relationships between tissue features and anisotropic intracellular conductivities that can be incorporated into tissue models for exploring fundamental mechanisms of cardiac arrhythmogenesis and their treatment.

## 2. Methods

All the procedures for animal surgery and tissue collection were approved by the IACUC of the University of Utah.

### 2.1. Animal model, microscopy, and image pre-processing

We applied image stacks acquired from cardiac tissues from the normal and MI heart in our prior work [16]. In short, surgery was performed on adult New Zealand White rabbits to occlude the left circumflex coronary artery and cause MI in the LV free wall. We also performed sham surgery on animals to serve as control for our studies. In the sham surgery, all procedures except the ligation of the coronary artery were performed. Three weeks after surgery, the heart was harvested for tissue collection. The excised heart was chemically fixed, and mid-myocardial slices parallel to the epicardium of the LV wall were processed for immunohistochemistry. The slices were labeled for Cx43 to mark gap junctions, α-smooth muscle actin (α-SMA) to identify myofibroblasts and smooth muscle cells, vimentin for fibroblasts and other non-myocytes, and DAPI to mark nuclei, respectively. We also used wheat germ agglutinin (WGA) to mark the glycocalyx and extracellular matrix.

We imaged the samples in 3D, using a laser scanning confocal microscope Leica TCS SP8 (Leica Microsystems, Wetzlar, Germany) with a 40x oil immersion objective. A 2D (xy) image with a size of 204 μm by 204 μm was acquired with a pixel size of 200 nm × 200 nm. Imaging was repeated using 200 nm steps in z-direction, yielding 3D image stacks of 176–300 slices. We avoided regions containing large blood vessels. We rotated the field of view to align myocytes with the y-axis of the image. The microscope was configured to apply linearly increasing excitation laser power with the depth of imaging to compensate for decreasing signal intensity (Leica Application Suite X). The MI images were obtained from regions within 1200 μm of the scar.

We described our methods for processing the 3D image stacks in detail previously [17,18]. In short, pre-processing included deconvolution with measured point spread functions of the microscope objective and depth-dependent attenuation correction based on the laser power increase during the acquisition. The thresholded WGA signal was post-processed using a voting filter to extract the outer sarcolemma. The distance map from the sarcolemma was used to generate segments of myocytes through a marker-based watershed workflow. The segments belonging to the same myocytes were interactively combined with a custom-written graphical user interface to generate segments representing individual myocytes. Similar to the segmentation of myocytes, capillaries were segmented using segments created from the thresholded WGA signal. After manually combining the segments representing the lumens of the capillaries, associated nuclei were included, and the segments dilated to include vessel walls. Fibroblasts and myofibroblasts were segmented in the residual space that was not classified as cardiomyocyte or capillary. As for the capillary segmentation, clefts were segmented by merging segments generated from the thresholded WGA signal.

### 2.2. Deep learning-based refinement of cardiomyocyte segmentation

While the processed WGA image stacks allowed us to reliably segment the extracellular space, further processing was necessary for accurate cardiomyocyte segmentation for the generation of models of conductivities of the intracellular space and intercellular coupling. Therefore, to correct individual cardiomyocytes in the previously generated segmentations, we developed a deep learning-based refinement approach.

A ground truth image set was obtained by extracting dilated contours from previously segmented images. The ground truth image set was split into a training, validation, and test data set using the ratio 3:1:1. The input image set consisted of random crops (size: 256 × 256 × 32 × 3; width × height × depth × channels; batch size of 4) of raw WGA, DAPI, and Cx43 images. Each tile was normalized per channel by dividing by its 95% quantile. We made use of chunked data storage within the HDF5 file format to load data on-the-fly. Tiles were extracted from random positions and fed into a modified variant of a 3D U-Net [19] predicting a binary two-pixel-thick mask of the cardiomyocyte boundaries. The used 3D U-Net architecture is shown in detail in Fig. S1. In short, convolution blocks consisted of two convolutional layers with 3 × 3 × 3 kernels, each followed by a rectified linear unit, followed by a batch normalization layer. Downsampling in the encoding path was done using 2 × 2 × 2 max-pooling operations. Upsampling with a factor of 2 × 2 × 2 in the decoding path was implemented with tri-linear interpolation operations instead of the originally used transposed convolution layers. We followed the scaling strategy of the convolutional filters of the original implementation. However, our first convolutional layer used 8 filters instead of 32. A sigmoid function was used to convert logits into probabilities. The network architecture had 1,021,937 trainable parameters in total.

Our implementation used the PyTorch framework [20]. We used the Adam optimizer with initial decay rates $\beta_{1}=0.9$ and $\beta_{2}=0.999$, and an initial learning rate ε=0.0004 [21]. We applied the native implementation of mixed precision training of the PyTorch package. Minimization was based on a loss function ℒ defined as the mean of the binary cross-entropy (BCE) loss ${ℒ}_{\text{BCE}}$ and the dice loss ${ℒ}_{\text{Dice}}$:

(1) $$ℒ=0.5\left({ℒ}_{BCE}+{ℒ}_{Dice}\right)$$

In initial tests, we observed that minimizing the summary loss function was more stable during training than only minimizing dice or BCE loss. Input tiles were augmented with random flips and elastic deformations during training. The training was stopped when the validation error did not improve over five epochs. Tile predictions were generated with 50% overlap and merged using Gaussian blending. The resulting boundary predictions were used to generate segments using the marker-based watershed workflow as described before. We generated an initial agglomeration of the segments by minimizing a MultiCut formulation [22]. We screened and corrected erroneous cardiomyocyte segments in our previous segmentations using a custom-developed graphical user interface. The user interface allowed for the further watershed-based generation of segments based on the boundary prediction, merging and unmerging of segments, and transferring of segments between segmentations.

### 2.3. Quantification of tissue features

We quantified segmented myocytes $\left(V_{myo}\right)$, vessels $\left(V_{vessels}\right)$, fibroblasts $\left(V_{fibro}\right)$, and myofibroblasts $\left(V_{myofibro}\right)$ as the fractional volume occupied by the respective segmentations in each image stack. The volume fraction of the ES $\left(V_{e}\right)$ was defined as the residual volume that was not segmented as cardiomyocyte, capillary, fibroblast, or myofibroblast:

(2) $$V_{e}=100\%-\left(V_{myo}+V_{fibro}+V_{myofibro}+V_{vessels}\right)$$

The volume fraction of segmented clefts $\left(V_{clefts}\right)$ was included in $V_{e}$. Our definition of fibrosis was based on its extracellular and cellular constituents, i.e. $V_{e}$, $V_{fibro}$, and $V_{myofibro}$. As described in detail in our previous work [16], the volume fraction of fibrosis $\left(V_{fibrosis}\right)$ was defined as the increase in these constituents in MI tissue compared with their mean in control tissue:

(3) $$V_{fibrosis}=V_{e,MI}+V_{fibro,MI}\;\;\;\;\;\;+V_{myofibro,MI}–mean\left(V_{e,control}+V_{fibro,control}+V_{myofibro,control}\right)$$

Processing of the Cx43 signal involved histogram-based thresholding with a threshold defined as mode plus 4.5 times standard deviations (SDs). Then, segmented myocytes were 3x dilated (26-neighborhood) to ensure that adjacent myocytes were in close connection. Subsequently, myocytes were ‘isolated’ with a one voxel layer separating adjacent myocytes, by removing voxels within the cardiomyocyte mask that would ‘connect’ two cardiomyocytes within a 26-neighborhood. A boundary around the segmented myocytes was generated by subtracting a 26-neighborhood dilation of the myocytes from the non-dilated myocytes. In this way, a one-voxel boundary containing the potential locations for computational gap junction elements was generated. To assign the gap junction elements, the processed Cx43 signal within 1 μm of the boundary was projected onto the aforementioned one-voxel layer. Each Cx43 voxel was projected to the nearest membrane voxel determined by its Euclidian distance. Out of the projected Cx43 voxels, only those voxels that fell on the membrane voxels shared by at least two myocytes were defined as gap junctions-containing voxels. We measured the volume fraction of gap junctions-containing voxels $\left(V_{gj}\right)$.

The stacks were analyzed for the orientation of myocytes and clefts. The average orientation of the largest 10 myocytes by volume was calculated from the eigenvectors of their second-order central moments [23]. The deviation of the orientation from the y-axis $\left(\Delta \text{l}\right)$ was quantified for all stacks. The normal to the largest interlaminar cleft plane and its deviation from the x-axis $\left(\Delta \text{n}\right)$ was also calculated. Stacks with weak alignment of microstructure, $\Delta \text{l>}10^{{}^{\circ}}$ or $\Delta \text{n>}30^{{}^{\circ}}$, were excluded from the analysis.

We extracted non-overlapping stacks from the processed image stacks, each covering 100 μm in x- and y-direction, and the full depth of the image stacks. The resulting 50 stacks were used in the computation of conductivities.

### 2.4. Computational setup for estimation of passive conductivities

Based on our prior work in calculating intracellular conductivities [24] and improvements presented in [25], we developed a computational setup to estimate intracellular electrical conductivity tensors of the control and MI tissues. We created models of the distribution of electrical conductivities in the intracellular space and intercellular coupling from the myocyte and Cx43 segmentations, respectively. These conductivity models had the same resolution and dimension as the underlying non-overlapping segmented stacks.

We used a cytoplasmic conductivity of 0.86 S/m [26]. Conductivities of gap junctions-containing voxel $\left(\sigma_{gj}\right)$ were derived from the 3D reconstructions of Cx43 signal. The signals were projected onto the segmented outer boundary of cardiomyocytes. The boundary voxels containing this projected signal were considered as the coupled Cx43 voxels. To determine the conductivity of a coupled Cx43 voxel, we established a relationship between the coupled connexin voxels between a pair of myocytes and the previously reported value of myocyte pair conductance. Hence, we calibrated the average number of coupled Cx43 voxels between all pairs of myocytes in control image stacks $\left(N_{gj,control}\right)$ to an average cell-cell pair conductance of 1.24 μS measured in normal myocytes [27]. For voxels with a length of l and a side surface area A, $\sigma_{gj}$ was calculated as

(4) $$\sigma_{gj}=\frac{1.24\;\mu S}{N_{gj,control}}\frac{l}{A}$$

Applying an electrical field along the long axis of the myocytes was the basis for the calculation of longitudinal conductivity $\left(\sigma_{myo,l}\right)$. Applying an electrical field across the long axis and along the plane of interlaminar clefts yielded the transverse conductivity calculation $\left(\sigma_{myo,t}\right)$. An applied electrical field normal to the interlaminar clefts led to the normal conductivity $\left(\sigma_{myo,n}\right)$. Therefore, Dirichlet boundary conditions of +1 V and −1 V were applied at the opposite ends of the simulation domain. We used a specialized finite difference approximation of Poisson’s equation to calculate the electrical potential $\varphi_{dir}$ for the given boundary conditions [28]:

(5) $$\nabla \cdot \left(\sigma \nabla \varphi_{dir}\right)=0$$

with the conductivity σ from the image-derived conductivity model.

We accounted for numerical issues arising from isolated elements, which were connected to neither positive nor negative electrodes. Therefore, we excluded these element that could not be reached by a region-growing algorithm seeded at the electrodes. The finite difference approximation of Poisson’s equation was solved as a linear system of equations using the C++ interface of PETSc [29]. The flexible generalized minimal residual (FGMRES) solver was utilized with the geometric algebraic multigrid (GAMG) preconditioner. We used a total size of the residual norm of less than 1e-10 as the convergence criterion of the solver.

Subsequently, to obtain the current density $J_{dir}$ within the domain, we applied finite differences to solve:

(6) $$J_{dir}=\sigma \nabla \varphi_{dir}$$

The directional conductivity $\sigma_{dir}$ was then calculated from the current density at each slice parallel to the electrodes averaged over the whole stack and the electrical field for given boundary conditions $E_{dir}$:

(7) $$\sigma_{dir}=\frac{mean\left(J_{dir}\right)}{E_{dir}}$$

### 2.5. Validation

We investigated our framework, using two approaches: First, to verify the accuracy of the numerical implementation of our estimation workflow, we estimated $\sigma_{myo,l}$, $\sigma_{myo,n}$, and $\sigma_{myo,t}$ in synthetic tissue models *with* brick-shaped myocyte, and compared it against an analytical solution. The error of the numerical estimation was <1e-10 S/m. Second, to investigate the effect of the size of stacks on the conductivities, we performed validation as described in the supplementary material. Briefly, we calculated conductivities for synthetic tissue models with brick-shaped myocytes to consider that some stacks contained myocytes that bridge an entire dimension or are truncated at stack borders.

### 2.6. Statistical analysis

MATLAB (version 2020a and higher, MathWorks, Inc., Natick, MA, USA) was used for statistical analyses of tissue features and to establish relationships with conductivities using linear regression. We evaluated the regression by the coefficient of determination (R^2^) and a p-value specifying the significance of the difference vs. a constant model. p < 0.05 was considered significant. We defined the effect size of relationships as weak if R^2^ < 0.3, moderate if R^2^ is between 0.3 and 0.5, and strong if R^2^ > 0.5. When the regression analysis of the conductivities with any of the features yielded a p < 0.05 only for control or only for MI, but p > 0.05 when combined, the R^2^ and p-values were reported separately. Separate R^2^ and p-values were also reported when the difference in R^2^ between control and MI was >0.1. Two-sample *t*-test was performed on the calculated conductivities and tissue features for the control and MI group. p < 0.05 was considered significant. Boxes in the boxplots represent interquartile range, and whiskers extend to 1.5 times the interquartile range beyond the boxes. Values for tissue features were reported as mean ± SD.

### 2.7. Visualization

The imaged signals were visualized in Fiji [30]. Volume reconstructions, potential distributions, current densities, and current density streamlines were visualized using Paraview [31]. Current density streamlines were seeded using spatially stratified random sampling at the plane corresponding to the positive electrode. 200 streamlines were visualized per figure.

## 3. Results

### 3.1. Confocal images and reconstruction of the control and MI tissue

**Fig. 1.**
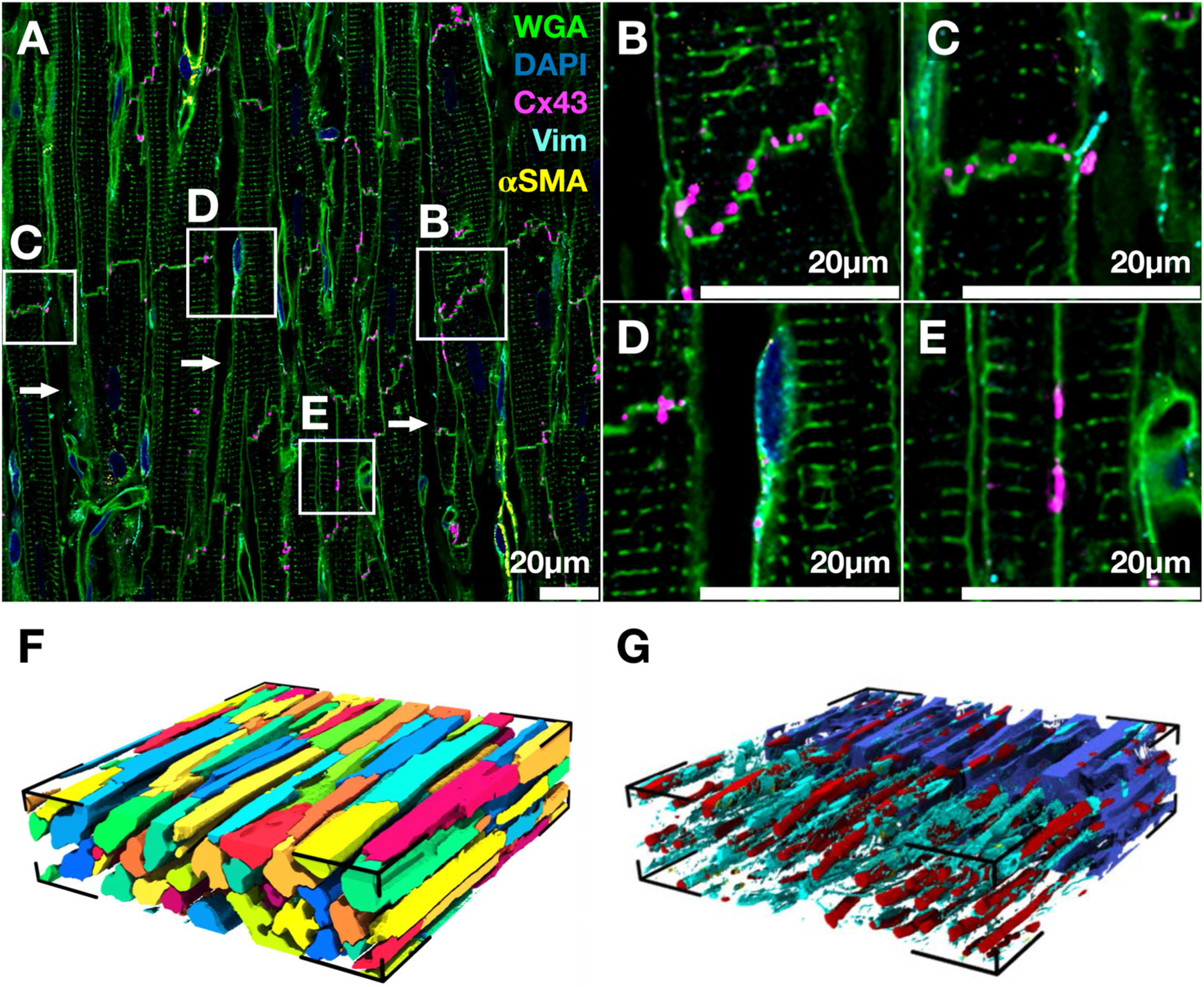
Composition and 3D reconstruction of myocardium from control animal. (A) Signals for WGA, DAPI, Cx43, vimentin, and α-SMA labeling are depicted in green, blue, magenta, cyan, and yellow, respectively, in a slice from an image stack. Arrows indicate interlaminar cleft spaces. Most Cx43 signal is found at end-to-end and side-to-side myocyte junctions. **(B**–**E)** Zoom-ins for the white boxes in (A) display representative microstructural features. **(B)** Cx43 labeling at the end-to-end junction of two myocytes. **(C)** End-to-end Cx43 labeling and adjacent vimentin-positive cell fragment in the interstitium. **(D)** End-to-end Cx43 labeling and vimentin-positive cell. **(E)** Side-to-side Cx43 labeling between two myocytes. **(F)** Reconstructed myocytes from the image stack. Myocytes were colored individually. The black line segments mark the edges and corners of the image stack. **(G)** Reconstructed non-myocyte contributors to the myocardium with ES in blue, vessels in red, and fibroblasts in cyan. The ES visualization is clipped in half for visibility of other tissue constituents.

Prominent features in an image stack of normal tissue labeled for WGA, DAPI, Cx43, vimentin, and α-SMA are highlighted in Fig. 1. ES, sarcolemma including transverse tubular system (t-system), and structures associated with capillaries were labeled by WGA (Fig. 1A). The long axes of myocytes were aligned with each other. Myocytes were densely packed. The Cx43 signal was present at myocyte ends and sides. Zoom-ins present examples for Cx43 arrangements. In Fig. 1B, the end-to-end junction between a pair of myocytes shows the characteristic staircase pattern, with Cx43 distributed throughout the sarcolemma at the junction. Another example for an end-to-end junction exhibits a sparser distribution of Cx43 and a vimentin-positive cell fragment (Fig. 1C). Another example shows a vimentin-positive cell adjacent to the myocyte membrane and with Cx43 labeling (Fig. 1D). Cx43 labeling was also present at lateral borders of myocytes, suggesting side-to-side coupling of myocytes in normal direction (Fig. 1E).

We calculated tissue features in the 3D reconstructions derived by using the refined segmentation approaches. $V_{myo}$ was 61.00% of the volume (Fig. 1F). The cross-sectional profiles of myocytes visualized in the x-z plane illustrate the dense packing with a characteristic arrangement of capillaries and clefts (Fig. 1G). The capillaries were also mostly aligned with the long axis of myocytes and $V_{vessels}$ accounted for 5.57% of the stack volume. Fibroblasts were present throughout the stack, with $V_{fibro}$ accounting for 2.91% of the stack volume. $V_{e}$ was 30.44%. Regions positive for α-SMA were negligible with $V_{myofibro}=0.08\%$ and not visualized. In this example image stack, $V_{gj}$ was 0.08% of the volume.

**Fig. 2.**
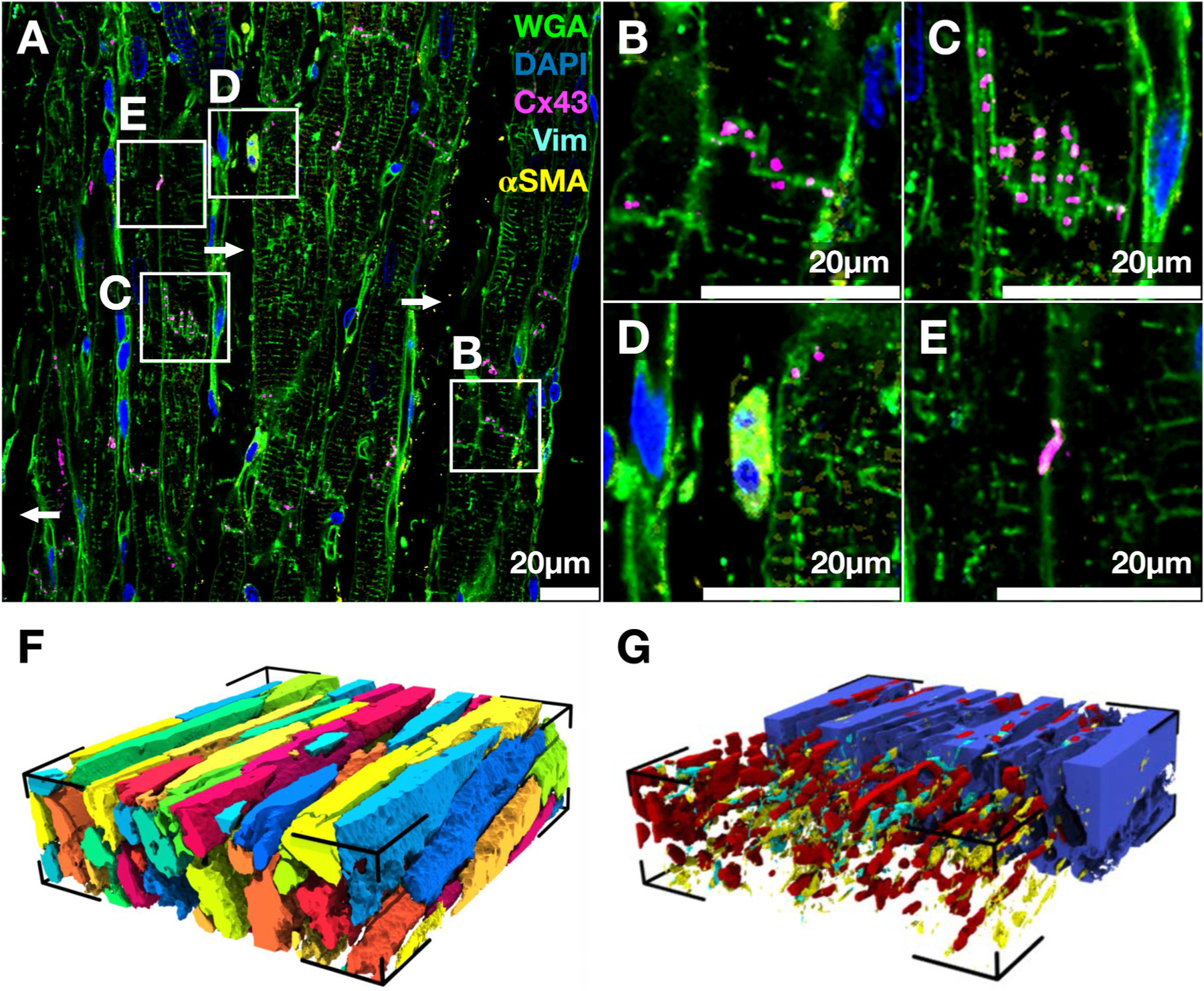
Composition and 3D reconstruction of myocardium from MI animal. (A) WGA, DAPI, Cx43, Vimentin, and α-SMA in green, blue, magenta, cyan, and yellow, respectively in a slice from MI image stack. Zooms into boxes in (A) depict **(B)** end-to-end Cx43 labeling between two myocyte pairs, **(C)** complex end-to-end junction between myocytes with Cx43 labeling, **(D)** α-SMA positive cell near a myocyte membrane, and **(E)** side-to-side myocyte coupling with Cx43 labeling. **(F)** Reconstructed myocytes from the image stack. **(G)** Reconstructed non-myocyte contributors to the myocardium with ES in blue, vessels in red, fibroblasts in cyan, and myofibroblasts in yellow.

Images from a tissue region at a distance of 250–750 μm to the infarct scar (Fig. 2A) revealed pronounced differences in the microstructural arrangement from that of normal tissue. For example, myocytes at the left side of the image were not parallel to those at the right side. Visual inspection also revealed expanded interlaminar clefts. Cx43 signal was more sporadic than in the normal tissue, reflected in a $V_{gj}$ of only 0.02% of the volume. Cx43 signal was present at the adjacent end-to-end membranes of the myocytes (Fig. 2B and C). The membrane at the intercalated disk displayed an intricate staircase pattern with which the Cx43 signal was aligned (Fig. 2C). Cells positive for both vimentin and α-SMA were found (Fig. 2D). We classified them as myofibroblasts. Cx43 was also observed on myocyte lateral borders, indicating side-to-side coupling (Fig. 2E). The reconstructed myocytes exhibited a less dense packing and larger cross sections than the normal tissue (Fig. 2F). $V_{myo}$ was 50.22%. ${\text{V}}_{\text{e}}$ was with 45.47% more pronounced vs. control (Fig. 2G). Capillaries were more fragmented and covered a smaller volume than in the control stack $\left(V_{vessels}=\;3.34\%\right)$. $V_{fibro}\left(0.25\%\right)$ and $V_{myofibro}\left(0.72\%\right)$ contributed marginally to fibrosis. Note that there were large variations of the volume fractions between the MI samples because of the heterogeneous remodeling in the BZ.

**Fig. 3.**
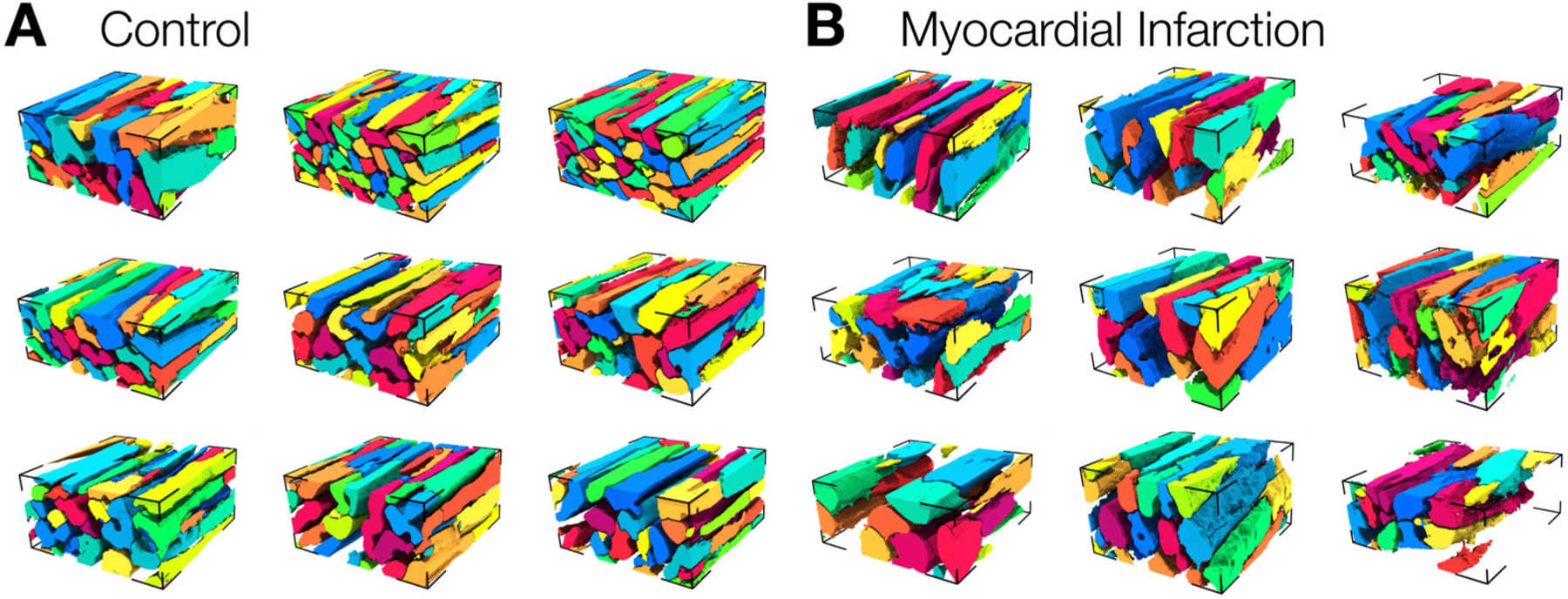
Representative myocyte reconstructions. (A) Reconstructions of myocytes from control image stacks of 100 μm in length and width with myocytes individually colored. The reconstructions illustrate the dense packing and alignment of myocytes. **(B)** Reconstruction myocytes from MI image stacks depict a larger variety including pronounced size differences and disarray among myocytes.

Stacks were created by subdividing the stacks such as those in Figs. 1 and 2 into non-overlapping sub-stacks with a width and length of 100 μm. Subsequently, we assessed the composition of these stacks and used them to derive conductivity models for the numerical calculations of electrical field and estimation of conductivity tensors. Representative reconstructions of myocytes from different stacks are visualized (Fig. 3). The visualization captures the variety in control (Fig. 3A) and MI stacks (Fig. 3B). The reconstructions demonstrate increased interstitial spaces between myocytes and their larger cross-section in MI stacks vs. control.

### 3.2. Potential and current density distribution in control and MI stacks

**Fig. 4.**
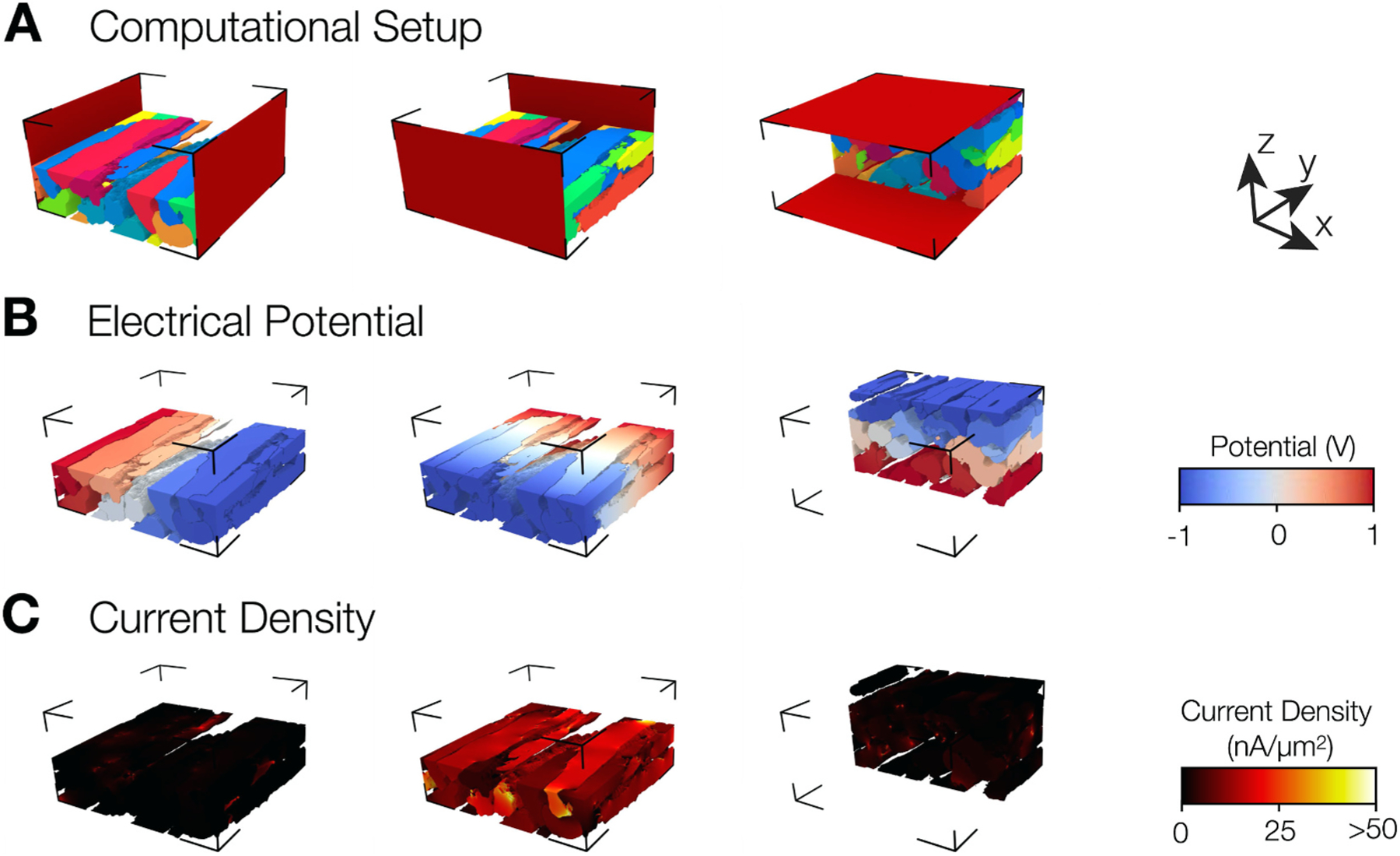
Electrode placement and field calculation in conductivity model derived from control image stack. (A) Reconstructed myocytes with electrodes visualized as red planes placed. Only half of the myocytes are shown. We applied electric fields along normal, longitudinal, and transverse directions. The orientation of arrows shows the correspondence to x, y, and z directions, respectively. **(B)** Potential distribution due to applied field with red, white, and blue corresponding to 1, 0, and −1V. **(C)** Current density distribution shows sparse regions of non-zero values in x and z directions in contrast to current density along y.

We applied Poisson’s equation for stationary electrical currents in models of the distribution of electrical conductivities in the intracellular space and intercellular coupling derived from the reconstructions. Solving the equation yielded intracellular electric potential fields along three orthogonal directions. Examples for the conductivity models, calculated distributions of electrical potentials and current densities are shown in Fig. 4A, B, and C, respectively. The normal, longitudinal, and transverse field application corresponding to the x, y, and z-axis are depicted in the left, middle, and right panels, respectively. Potentials due to field application along the normal direction displayed large gradients at the side-to-side coupling between myocytes and negligible gradients within each myocyte. Very few locations in the intracellular space exhibited appreciable current density. Potentials caused by field application along the longitudinal direction, i.e. the long axis of myocytes, displayed a gradual change of potential over most of the regions within myocytes. A larger gradient was observed here also at locations of coupling between two myocytes. The current density was higher in longitudinal than normal direction. Potentials due to field application along the transverse direction showed a similar gradient of potential as for field application in the normal direction. The resulting current density was heterogenous and much smaller than that for field application along the longitudinal direction.

**Fig. 5.**
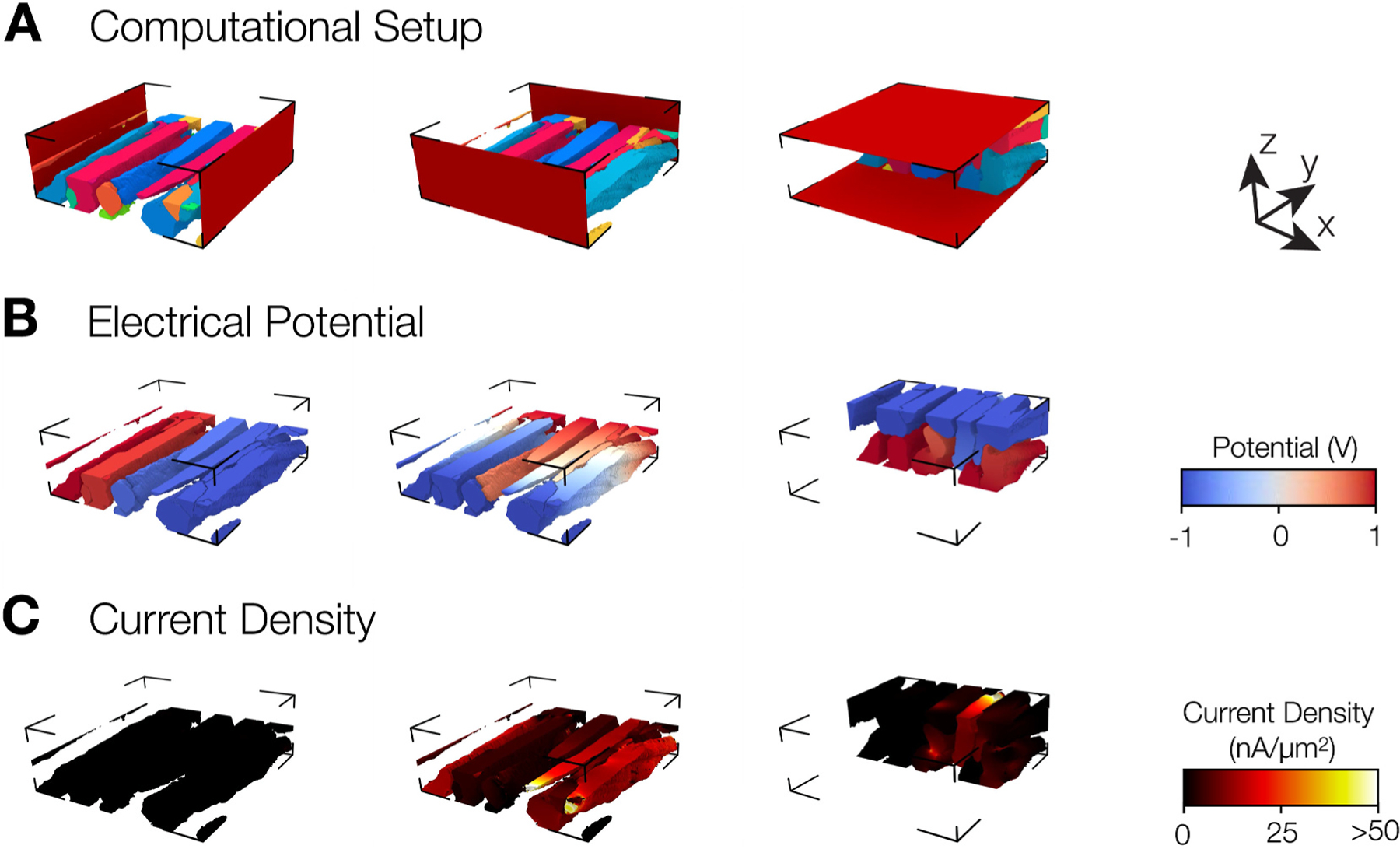
Electrode placement and field calculation in conductivity model derived from MI image stack. (A) Reconstructed myocytes with electrodes for fields applied along x, y, and z directions. In part, a loss of direct connection occurred between myocytes as well as between electrodes and myocytes due to large ES. **(B)** Potential distribution through the reconstruction along the three axes. **(C)** Current density visualized on myocytes.

A representative MI stack is presented in Fig. 5. Visualization of the potential (Fig. 5B) and current density distribution (Fig. 5C) along the three orthogonal directions revealed notable differences from those of the control stack (Fig. 4B and C, respectively). The potential changes in normal and transverse directions were less continuous than in control. Current densities for normal and transverse field application were much lower than for longitudinal field application. The current density for longitudinal and transverse field application exhibited more heterogeneity vs. control (Fig. 5C vs. 4C).

**Fig. 6.**
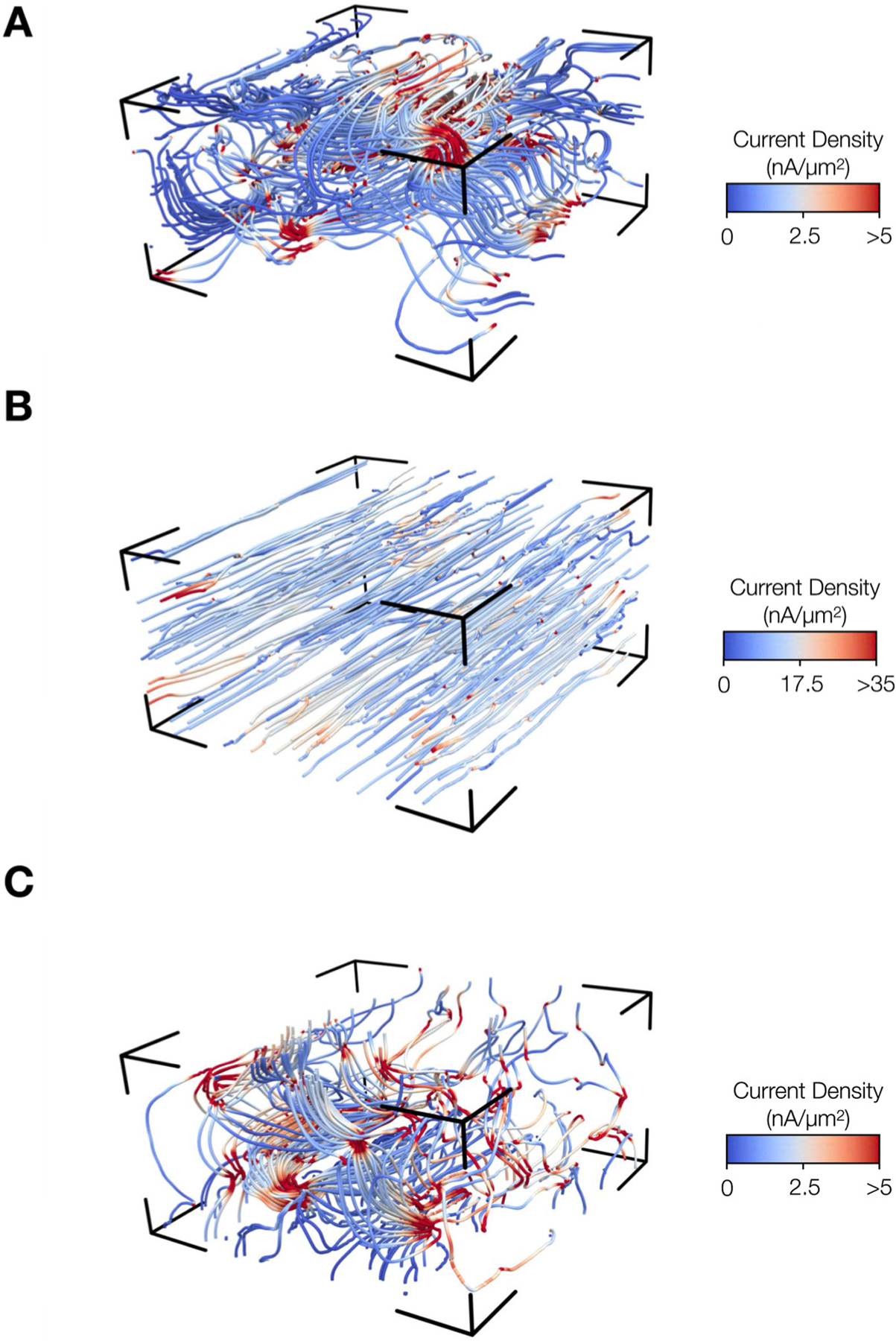
Streamline visualization of simulated current densities in myocardium. Current density streamlines in a control stack for the electric field applied along **(A)** normal, **(B)** longitudinal, and **(C)** transverse direction, i.e. x, y, and z direction, respectively. The streamlines were seeded at the center of the stack on a plane parallel to the electrodes and trace the paths of current flow. A high density of streamlines identifies locations with a high current density.

Fig. 6 visualizes the current densities of the control stack Fig. 4 as streamlines along the normal (Fig. 6A), longitudinal (Fig. 6B), and transverse (Fig. 6C) directions. The streamlines trace the current flow in the tissue related to the potentials applied to the electrodes. The locations of high current density are marked by convergence of the streamlines. The streamlines in longitudinal direction were mostly aligned perpendicular to the electrode planes. The streamlines illustrate the limited intercellular coupling for normal and transverse directions, and hence a higher resistance and reduced current flow. Less frequent locations of higher current density were present in the simulation of the normal vs. the transverse field, reflecting differences in inter-myocyte coupling.

### 3.3. Tissues features and conductivities distinguish MI from control

**Fig. 7.**
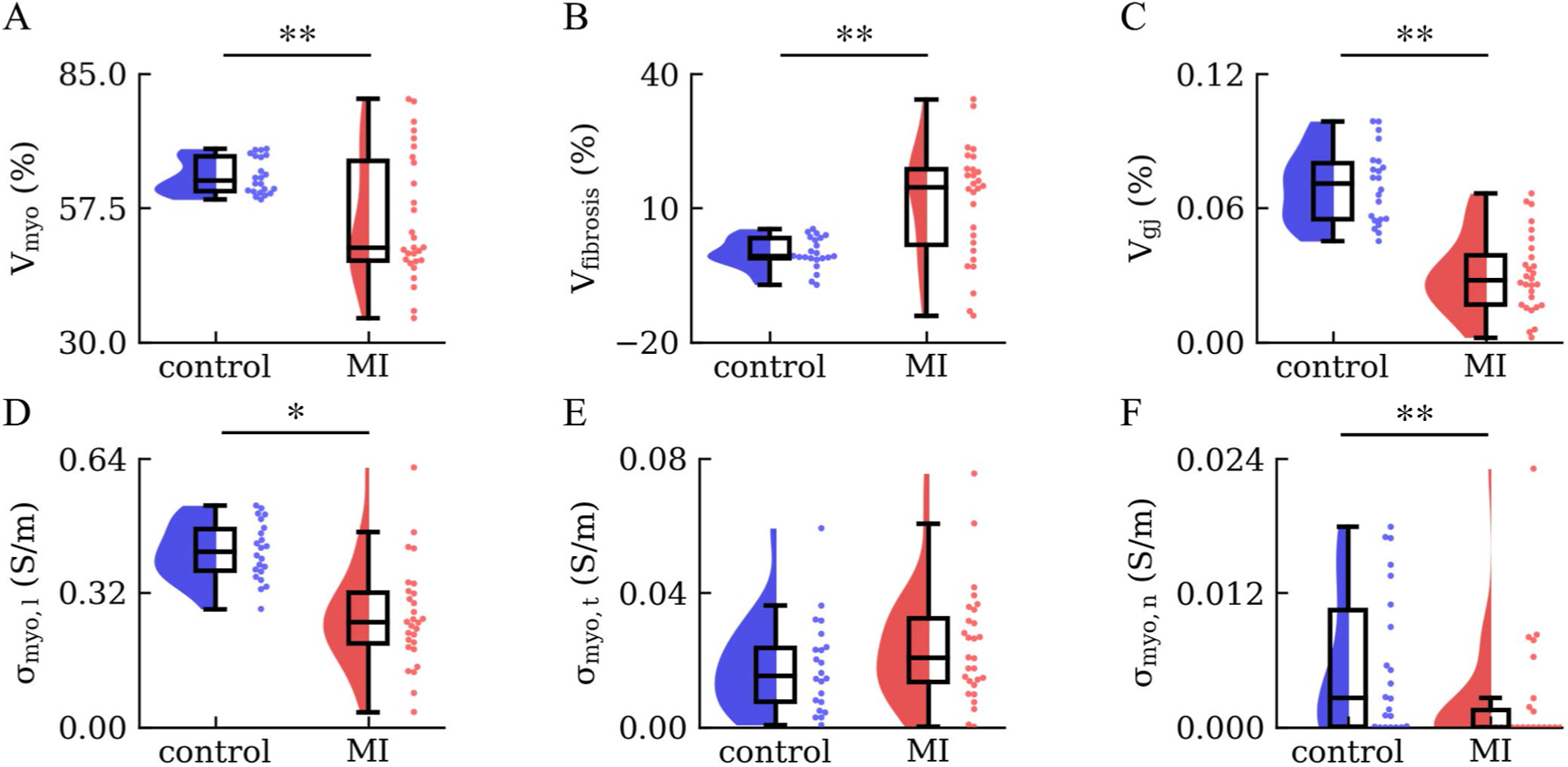
Statistical analyses of tissue features and conductivities. We used violin, box, and strip plots. **(A)** $V_{myo}$, **(B)** $V_{fibrosis}$, and **(C)** $V_{gj}$ differed in control (blue, n = 22) and MI (red, n = 28) samples. **(D)** $\sigma_{myo,l}$ was decreased in MI samples. **(E)** The difference of $\sigma_{myo,t}$ for control and MI samples was marginal. **(F)** $\sigma_{myo,n}$ was decreased in MI samples. **: p < 0.01, *: p < 0.05.

We compared the tissue features and conductivities in the MI and control groups (Fig. 7). $V_{myo}$ was higher in control than in MI (64.04 ± 3.66 vs 55.26 ± 12.98%, Fig. 7A). $V_{fibrosis}$ was increased in MI vs. control (11.27 ± 12.65 vs 0.00 ± 3.50%, Fig. 7B). $V_{gj}$ was higher in control than MI (0.07 ± 0.02 vs 0.03 ± 0.02%, Fig. 7C).

In MI, $\sigma_{myo,l}$ was lower than in control (0.264 ± 0.121 vs. 0.419 ± 0.069 S/m, Fig. 7D). Also, $\sigma_{myo,n}$ was lower in MI than in control (0.002 ± 0.005 vs. 0.006 ± 0.007 S/m, Fig. 7F). However, $\sigma_{myo,t}$ was not significantly different between the two groups (0.024 ± 0.017 vs. 0.018 ± 0.014 S/m, Fig. 7E).

### 3.4. Tissue microstructure affects conductivities

**Fig. 8.**
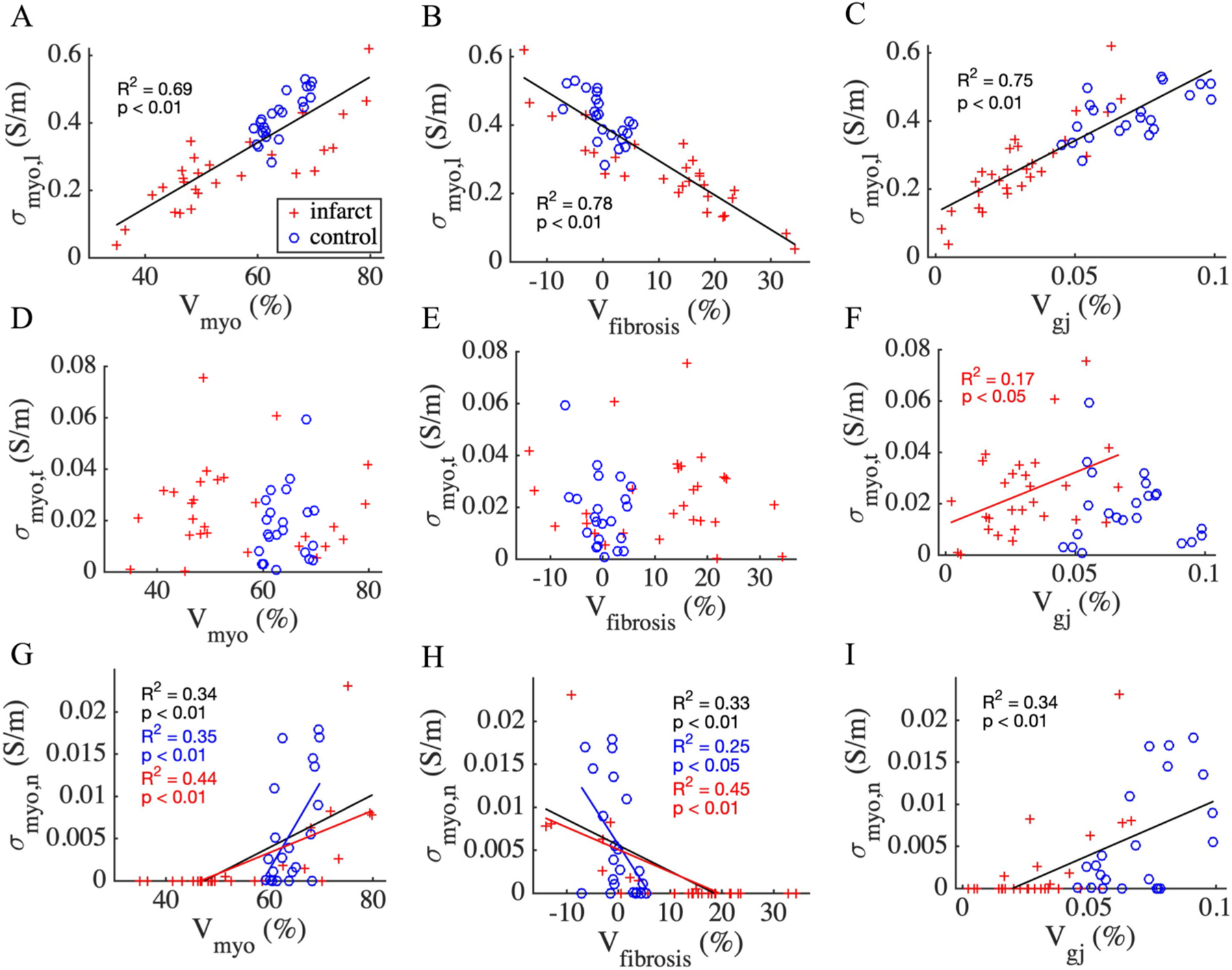
Regression analyses of tissue features and conductivities. Linear regression lines in black, blue, and red, wherever present, applied to all samples, control samples (n = 22) alone, and MI samples (n = 28) alone, respectively. **(A)** The relationship of $\sigma_{myo,l}$ with $V_{myo}$ was strongly positive. **(B)** $\sigma_{myo,l}-V_{fibrosis}$ was strongly negative. **(C)** $\sigma_{myo,l}-V_{gj}$ was strongly positive. $\sigma_{myo,t}$ did not exhibit relationships with **(D)** $V_{myo}$ and **(E)** $V_{fibrosis}$. **(F)** $\sigma_{myo,t}$ had a weak positive relationship with $V_{gj}$ for MI samples alone. **(G)** $\sigma_{myo,n}$ increased with increasing $V_{myo}$ and **(H)** decreased with increasing $V_{fibrosis}$. **(I)** $\sigma_{myo,n}$ increased as $V_{gj}$ increased.

We also explored relationships of conductivities with the tissue features (Fig. 8 & Table S1). Linear regression revealed strong and moderate positive relationship of $V_{myo}$ with $\sigma_{myo,l}$ (Fig. 8A) and $\sigma_{myo,n}$ (Fig. 8G), respectively. However, we did not find such a relationship between $V_{myo}$ and $\sigma_{myo,t}$ (Fig. 8D). The relationship between $\sigma_{myo,n}$ and $V_{myo}$ was moderate with increased R^2^ when considering MI samples and control samples separately (R^2^ = 0.34 vs. R^2^ = 0.44 and 0.35, respectively).

The negative relationship of $V_{fibrosis}$ with $\sigma_{myo,l}$ and $\sigma_{myo,n}$ was strong and moderate, respectively (Fig. 8B, H). While the MI samples alone displayed a moderate relationship between $\sigma_{myo,n}$ and $V_{fibrosis}$ (R^2^ = 0.44), the control samples alone exhibited only a weak relationship (R^2^ = 0.25) (Fig. 8H). $V_{gj}$ was a strong modulator of the conductivities. In particular, $\sigma_{myo,l}$ increased with increasing $V_{gj}$ for all samples (R^2^ = 0.75) (Fig. 8C). Also, the MI samples displayed such a strong relationship (R^2^ = 0.69). The relationship between $\sigma_{myo,n}$, and $V_{gj}$ was moderate for control and MI (Fig. 8I). Though $\sigma_{myo,t}$ did not change overall with $V_{gj}$, there was a weak effect of $V_{gj}$ on $\sigma_{myo,t}$ for the MI samples alone (Fig. 8F). We did not find such a relationship in the control samples alone.

From our analysis of the effect of stack size on conductivities, we found that stack lengths equal to or larger than 100 μm resulted in longitudinal conductivity values within 10% of the mean conductivity calculated at a stack length of 400 μm (Supplementary text and Figs. S2–S6).

## 4. Discussion

In this study, we presented a framework for quantitative, microscopy-based estimation of anisotropic intracellular conductivity tensors of cardiac tissues and applied it to compare conductivities in LV tissue in control and MI animals. We also provided a detailed quantification of the composition of LV tissue in control and MI animals. We established insightful relationships between the anisotropic conductivities and tissue features. The developed framework can be applied for estimation of conductivity tensors and establishing conductivity-microstructure relationships in other species and for other cardiac tissues and diseases. Examples for other applications include fibrotic atrial tissues and hypertrophic ventricular tissues in atrial fibrillation and heart failure, respectively.

Various approaches have been used in the past to determine the conductivities in the myocardium. A recent review comprehensively compares the conductivities available from measurements using electrodes, image-based simulations, and idealized models [12]. Each of these approaches has its merits and limitations. We summarized prior reports on intracellular conductivities of ventricular tissue in various species in Table 1. Measurement with electrodes that sample the potential distribution on tissue surfaces and within the tissue is the most common approach, but it does not directly provide extracellular and intracellular conductivities. Application of the measurements in a theoretical conduction model, e.g., a cable model, is required to derive the conductivities of the two domains. Limitations of the measurements are related to the complexity of the tissue geometry and microstructural arrangement that are difficult to cover with earlier theoretical conduction models. Reported conductivities, independent of their measurement methods, even within the same species, displayed a large spread (Table 1).

In our approach, we utilized an image-based reconstruction of tissue to describe the two domains of the tissue. These reconstructions have the unique benefit of integrating complex microstructural representations and arrangements, specifically including myocyte geometries, their gap junctions, and interlaminar clefts, which all incontestably impact myocyte conductivities. Further, our approach enables us to investigate myocyte conductivities at a microscopic scale of 100 μm, which is close to commonly used edge lengths in computational studies of cardiac conduction. This microscopic view offers a novel perspective compared to previous experimental studies, which mostly approached conductivities macroscopically, e.g. by investigating the average conductivity within a long segment of papillary muscle [32–34] or trabeculae [35, 36]. However, local heterogeneities in conductivities may be vital to explain arrhythmic events [37], and therefore investigating the distribution of conductivities may be equally of interest as averaged conductivities.

We calculated $\sigma_{myo,l}$ of 0.419 ± 0.069 S/m in our control rabbit LV myocardium, which was close to prior electrode-derived measurements in rabbit papillary muscle [32,33]. While other electrode-derived measurements in rabbit RV papillary and septum are also close, but higher than our estimation, imaging-derived estimations were lower (Table 1). Also, electrode-derived measurements from canine, bovine, and porcine tissues were lower. Many of these earlier measurements had considerable differences in the experimental setup and assumptions used to derive conductivities [34,39].

Prior measurements of $\sigma_{myo,t}$ were fewer and more varied. The lowest and highest measurements differ by a factor of 20 (0.003 vs. 0.06 S/m). Our result of 0.018 ± 0.014 S/m is similar to measurements in bovine RV trabeculae [35]. We did not find measurements of $\sigma_{myo,t}$ from rabbit LV. In a computer modeling study [43], the small $\sigma_{myo,t}$ is explained by the lack of gap junctions along the lateral sides of myocytes in the tissue model. Many studies assumed the same value for $\sigma_{myo,t}$ and $\sigma_{myo,n}$. However, it is now established that these are distinct [14]. Calculations of $\sigma_{myo,n}$ are sparse and also have an order of magnitude variation. We obtained $\sigma_{myo,n}=0.006\pm \;0.007\;S/m$, which is within the range of prior estimates.

While intracellular conductivities of myocardium in the MI heart have not been previously reported, it is obvious that the described remodeling in tissue microstructure strongly impacts these conductivities. A few studies reported the relative changes in conductivity due to acute ischemia. Tissue conductivity decreased acutely and after prolonged ischemia in rabbit LV tissue [45]. While the extracellular conductivity $\sigma_{e}$ decreased in ischemia, no significant change was found in $\sigma_{myo}$ [32]. In rabbit papillary muscles after 15 min of ischemia, $\sigma_{myo}$ rapidly decreased three-fold [33]. However, these short-term changes are not comparable to our MI model and the described tissue remodeling.

Modeling and simulation studies predicted changes in bidomain conductivities due to microstructural changes [42,43]. Computational simulation of electric fields in a geometric model of the myocardium showed decreased $\sigma_{myo,l}$, and $\sigma_{myo,t}$ with decreased cell-to-cell gap junction resistance [43]. Varying geometrical features also revealed that while increasing myocyte cross-sectional area did not affect $\sigma_{myo,l}$, it produced a positive linear relationship with $\sigma_{myo,t}$ [46]. Abnormally high myocyte cross-sectional area was also shown to be more prone to conduction block, discontinuous propagation and reduced longitudinal CV [11]. A model that varied $V_{e}$ and calculated conductivities reflected changes of effective bidomain conductivities with changes in $V_{e}$ [47].

A difficulty in the comparison of our conductivity estimations to prior measurements is related to the definition of the reference coordinate system of the conductivity tensors. In our work, the reference coordinate system was defined by visual inspection of tissue microstructure before imaging. Hence the tensor coordinate axes were aligned to our image coordinates. In prior work, the reference coordinate system was often based on a coordinate system assigned to the ventricles. Many times, however, these do not align with the local coordinates of the tissue conductivity tensors. This might explain the variations observed in Table 1.

A focus of our work was the assessment of variation of intracellular conductivities with the tissue features quantified from microscopic images. We found that $\sigma_{myo,l}$ strongly increased with increasing $V_{myo}$ and increasing $V_{gj}$. Within the control samples, $\sigma_{myo,l}$ varied from 0.28 to 0.53 S/m, which was explained by variation in $V_{myo}$ and $V_{gj}$. This is also largely consistent with the variation seen in the literature (Table 1). The larger variation of 0.04–0.62 S/m observed in MI BZ samples was also explained by the changes in $V_{myo}$ and $V_{gj}$. This relationship that holds across normal and diseased tissue is useful to guide parameterization of cardiac tissue models. We also found that $\sigma_{myo,n}$ increased moderately with increasing $V_{myo}$ and $V_{gj}$. However, $\sigma_{myo,n}$ was affected strongly by clefts and was 0 S/m in most samples, although there was a steep increase of $\sigma_{myo,n}$ up to 0.018 S/m in control samples with increasing $V_{myo}$. The increase of $\sigma_{myo,n}$ in MI BZ was more gradual. Though we did not find such relationships with the tissue features for $\sigma_{myo,t}$, its variation from 0.003 to 0.059 S/m in control tissue samples is consistent with the range of the previously published values (Table 1). The heterogeneity of quantified tissue features as well as conductivities are notable in the MI samples indicating the heterogeneity of remodeling in the BZ.

In prior work, parameters of computational models of cardiac tissue electrophysiology were often tuned to match measured conduction velocities (CVs) to those simulated [4,48,49]. However, this tuning of homogenized conductivities does not directly reflect the effects of fibrosis, connexin lateralization, and other changes that happen in microstructural remodeling. Moreover, tissue conduction models of diseased tissue have been developed by scaling of conductivity measurements of normal tissue due to the lack of measurements in diseased tissue [50]. Quantification of fibrosis observed in different diseased tissues can be used to parameterize the conductivity over the border zone in a non-binary manner from the relationships established in this study. Since 100 μm is a common edge length of computational elements used in the simulation of cardiac electrophysiology, element-wise parameterization of the domain based on these results is possible.

Major assumptions used in our calculation are related to the conductance of gap junctions between myocyte pairs and the conductivity of cytoplasm. The conductance between a myocyte pair was chosen to be 1.24 μS from measurements in normal rabbit ventricular myocytes [27]. It is well-established that Cx43 is the dominant protein of gap junctions in the rabbit LV [9]. Comparison of patch-clamp conductance measurement to confocal microscopic images of Cx43 showed a constant conductance per gap junction area [51].

For our simulations, we set the cytoplasmic conductivity to 0.89 S/m based on measurements from different experimental methods in atrial myocytes of guinea pigs [26]. The study performed the measurements through three independent techniques yielding consistent results. A previous study by the same authors in the frog ventricle that reported a much lower value was found to be error-prone due to the dependence of tissue resistance on microelectrode resistance [52]. A low value with anisotropy was calculated and used in modeling studies [53]. Consistent with this was a lower value of 0.17 S/m reported at room temperature in rat ventricular myocytes [54]. Another measurement of cytoplasmic conductivity in guinea pig ventricular myocytes was 0.92 S/m [55]. This study also showed that the cytoplasmic conductivity did not change in hypertrophy. Overall, cytoplasmic conductivities remain ill-defined and measurements for different cell types and species are lacking. Further studies are needed to quantify the impact of subcellular organelles on the conductivity of myocyte cytoplasm and its modifications in disease.

Our analyses showed that a myocyte is connected via gap junctions to 4.37 ± 0.61 other myocytes in control samples vs. 2.98 ± 1.13 in MI. However, the length of 100 μm of our stacks limited coverage of all the neighbors of each myocyte. Though the numbers of connected myocytes were underestimated, they show the reduction in connectivity from control to MI. Light microscopy of normal canine LV subepicardial tissue yielded an average of 9.1 neighboring myocytes connected to a myocyte through intercalated disks [56]. In our previous confocal image-based reconstruction of rabbit mid-myocardial tissue, we determined the number of neighboring myocytes to be 9.4 ± 0.5 and 4.3 ± 0.5 in an example control and MI stack, respectively [18]. However, our measurement was based on proximity and might have included neighboring myocytes that were not coupled through gap junctions. A study on canine LV tissue indicated that the number of myocytes connected through intercalated disks to a myocyte went down from 11.2 in normal to 6.5 in MI BZ of [57]. Hence, there is agreement in the reduction of neighboring myocytes and hence the number of coupled myocytes in the remodeled MI tissue vs. normal tissue.

In this work, we only considered the electrical coupling between myocytes. However, studies suggested coupling of fibroblasts, myofibroblasts, and macrophages with myocytes in disease [58–62]. The fraction of these non-myocytes that couple with myocytes and the variation in the coupling strength remains unquantified in normal and diseased myocardium. Our reconstruction of fibroblasts and myofibroblasts and their remodeling in the MI BZ provides a basis for modeling their effects on the conductivities of normal and diseased tissue once their electrical coupling with myocytes is quantified.

Interlaminar clefts are a major feature of the myocardial microstructure in rabbit and other species [16,18,63]. Accurate characterization of these clefts is complex, especially in diseased tissue. Fibrosis and the resulting expansion of interstitial space confound identification of the clefts. We found that $\sigma_{myo,n}$ is strongly affected by the presence of clefts. Many of our conductivity models yielded $\sigma_{myo,n}$ of 0 S/m due to the complete absence of electrical connection along the direction normal to the cleft planes since the clefts act as barriers in the inter-myocyte space. Even the presence of narrow clefts eliminates the connection since the presence of Cx43 signal on the myocyte membranes along the clefts is negligible. Hence, the possibility of myocyte to non-myocyte electrical coupling that can bridge across the cleft is low. In addition to the impact on anisotropy of the conductivity, the clefts have been found to play a role in the electrical activation of myocardium during defibrillation [64]. Whether the expanded cleft spaces in remodeled tissue affect this function is not yet clear. Moreover, the higher collagen deposition in existing clefts due to disease was also not quantified.

The orientation of the interlaminar clefts and myocytes affects the calculation of the anisotropic conductivities. We minimized the impact of rotation by excluding stacks that had large deviations of myocytes or clefts from the coordinate axes. Differences of Δl or Δn between the control and MI samples were marginal (Fig. S7). Our results indicated weak effects of Δn on $\sigma_{myo,t}$ and $\sigma_{myo,n}$ and Δl on $\sigma_{myo,l}$, $\sigma_{myo,t}$, and $\sigma_{myo,n}$. It is important to note that the coordinate system used in many measurements of conductivity tensors as well as CVs from macroscopic tissue is different from our approach based on microstructural orientation of myocytes and cleft planes. Transverse and normal direction in literature were often defined assuming a plane parallel to the epicardium or endocardium and transmural to the ventricular wall.

A limitation of our approach is related to 3D confocal microscopy of cardiac tissues at high resolution. Though we calculated the conductivity and characterized the tissue with a size of 100 μm in the longitudinal and normal direction, a similar extent in the transverse direction was not possible due to limitations in imaging. Increasing imaging depth to more than 100 μm while preserving the resolution and the sample’s integrity may be achievable through advances in tissue clearing and microscopy.

## 5. Summary

This is the first study comparing intracellular anisotropic conductivity tensors of normal cardiac tissues to remodeled tissue in the MI heart. Together with our prior work on estimation of extracellular conductivities [16], we provide a set of anisotropic bidomain conductivities of the myocardium in the control and MI heart. Our analyses revealed relationships between conductivities and remodeling of myocytes, gap junctions, ES, and fibrosis. Importantly, we established relationships between intracellular conductivities and the degree of fibrosis. Our framework can be directly applied to establish these relationships for other species, cardiac tissues and diseases. Future work could establish a comprehensive microstructure-based understanding of intracellular conductivities in the normal and diseased heart. Furthermore, the framework can be applied to estimate intracellular conductivities in non-cardiac tissues with intercellular electrical coupling. We suggest that our work supports the further development and translation of cardiac modeling and simulation. The relationships established in this study provide guidance for specifying conductivities in computational models of cardiac tissue electrophysiology based on the tissue features. The relationships can be applied to computational modeling of normal and diseased tissue. In addition, we suggest that the reported ranges and distributions of estimated conductivities provide a framework for assessing the predictive power of computational modeling and simulation of cardiac electrophysiology using uncertainty quantification.

## Supplementary Material

Supplemental Text and Table

Supplemental Figures

## Figures and Tables

**Table 1:** Overview of reported intracellular conductivities in ventricular cardiac tissue from various species. Normalized conductivities are conductivities calculated with the ratio of longitudinal to transverse space constant = 2, $\sigma_{myo,t}=\;0.035\;\;S/m$, as proposed in [38]. α is the ratio of intra- to extracellular longitudinal conductivity. The value indicated with ^a^ was derived using an extra- to intracellular space ratio of 1:4 as in Kleber et al. [33] instead of the originally used 1:3 ratio in [34].

Method	Species	Tissue	Intracellular Conductivity (S/m)			Reference
			$\sigma_{myo,l}$	$\sigma_{myo,t}$	$\sigma_{myo,n}$	
Electrode-derived	Bovine	RV trabeculae	0.17	0.019		Clerc [35]
Electrode-derived	Bovine/Ovine	RV trabeculae	0.16			Weidmann [36]
Electrode-derived	Canine	LV	0.28	0.026		Roberts et al. [39]
Electrode-derived	Canine	LV	0.34	0.06		Roberts and Scher [40]
Electrode-derived	Porcine	LV	0.35	0.04	0.01	Trew et al. [41]
Electrode-derived	Rabbit	RV papillary	0.60			Kleber and Riegger [34]
			0.45^a^			
Electrode-derived	Rabbit	LV papillary	0.48			Cascio et al. [32]
Electrode-derived	Rabbit	LV papillary	0.52			Kleber et al. [33]
Numerical model (imaging-derived)	Rabbit	LV	0.07	0.004	0.003	Bauer et al. [24]
Numerical model (idealized)	Murine	LV	0.14	0.003		Hand et al. [42]
Numerical model (idealized)			0.16	0.005		Stinstra et al. [43]
Normalized conductivities (α = 1.0)	Porcine	LV	0.24	0.035	0.008	Johnston [38]
Normalized conductivities (α = 0.6)	Porcine	LV	0.19	0.035	0.008	Johnston et al. [44]
Normalized conductivities (α = 1.6)	Porcine	LV	0.31	0.035	0.008	Johnston et al. [44]
**Numerical model (imaging-derived)**	**Rabbit**	**LV (control)**	**0.42**	**0.018**	**0.006**	**This study**
**Numerical model (imaging-derived)**	**Rabbit**	**LV (MI)**	**0.26**	**0.024**	**0.002**	**This study**
