# Supplementary material for: Confocal microscopy-based estimation of intracellular conductivities in myocardium for modeling of the normal and infarcted heart
Source: Comput Biol Med. Author manuscript; Available in PMC 2023 May 18. (PMC10195095; doi:10.1016/j.compbiomed.2022.105579)
Supplement: Supplemental Text and Table [file NIHMS1893710-supplement-Supplemental_Text_and_Table.docx]

**Supplemental Material**

# Confocal Microscopy-Based Estimation of Intracellular Conductivities in Myocardium for Modeling of the Normal and Infarcted Heart

**Joachim Greiner, Aparna C Sankarankutty, Thomas Seidel, Frank B Sachse**

**Supplemental Methods and Results

On the Dependence of the Calculated Myocyte Conductivity on Model Volumes**

The homogenized passive myocyte conductivity tensor is dependent on the volume in which the homogenized tensor is determined. If one aims to homogenize cardiac tissue into “representative” values, the question of how large the volume in which one homogenizes is critical and will lead to different results which answer different questions. There are vast differences in the tensor values when a small region within a cardiomyocyte (cytoplasmic conductivity; comparatively high conductance) and a small region that encapsulates gap junctions (comparatively low conductance) coupling two myocytes are compared. Such small regions may not be of much practical relevance, as one likely draws from a binary distribution of ‘high conductivity’ and ‘small conductivity’. On the other hand, a ‘too large’ region may ignore tissue heterogeneity, which is potentially a key characteristic of cardiac tissue, especially when investigating cardiac arrhythmia. Thus, depending on the volume of the studied region of interest, one has to choose a trade-off between local resolution and the ability to reflect the tissue behavior macroscopically. In the main studies of this paper, we investigated model volumes similar to those commonly used for computational elements in simulation studies (Fig. S2). The coordinate system of the *confocal microscopy-based* model stacks was aligned with the cardiomyocyte, sheet orientation, and sheet normal.

Here, we assessed with the help of a *synthetic* model the expected heterogeneity of the longitudinal passive myocyte conductivity. Our model assumes fixed cardiomyocyte dimensions, passive conductivities, and gap junction distributions. We established and quantified the trade-off of interest-dependent heterogeneity solely based on the investigated volume in a computational setup with the same dimensions as used in the main manuscript (102.4µm x 102.4µm x 47.4µm). The z-dimension was determined by calculating the mean value of all conductivity model stacks. We constructed a *synthetic model of cardiomyocytes connected by gap junctions*. In brief, we generated brick-shaped cardiomyocytes (length: 100µm, width: 20µm, height: 15µm) with one voxel-thick, randomly placed gap junctions with different probabilities for coupling side-side (p_side/side_ = 0.05%) and end-end (p_end/end_ = 0.25%). To fill the volume with cardiomyocytes, one prototype cardiomyocyte was created, and then placed with different x- and y- offsets throughout the volume (Fig. S3). To account for randomness, we sampled three different realizations of different gap junction distributions and geometrical cell offsets for these sets of experiments. We sampled each of these volumes at 0, 10, 20, 30, 40, 50, 60, 70, 80, and 90 µm length offsets (cropping offsets) along the cardiomyocyte axis (Fig. S4). Notably, the whole setup is periodic with a length of 100.4µm (cardiomyocyte length added to two voxel layers of gap junctions).

The subsequent conductivity estimation was executed as described in the main paper with the exception of the normalization of the gap junction values. The conductance of gap junctions was normalized to equal resistances across the gap junctions and the cytoplasm of the cardiomyocyte (Fig. S5).

The resulting heterogeneities for different stack lengths and, therefore, crop volumes, are shown in Fig. S6. Compared to the mean conductivity value at a stack length of 400µm, stack lengths smaller than the cardiomyocyte length of 100µm had larger heterogeneities and were larger (160±16%, 134±13%, 117±11% for stack lengths of 25, 50, and 75µm, respectively) than stack length equal or larger than the cardiomyocyte length (106±3%, 105±4%, 104±3%, 103±3%, 102±1% for stack lengths of 100, 125, 150, 175, and 200µm, respectively).

Notably, we expect the heterogeneity originating from cropping to be independent of the measurement method. E.g., the design of studies using microelectrode arrays for conductivity estimation will also consider the trade-off between local and global resolution/ representation. Our paper is focused on applications in computational electrophysiological studies. Thus, we orient ourselves to a commonly used voxel length of around 100µm. However, given the results here, it is likely that observed variations between different crops from one conductivity model stack reflect more microstructural features (myocardial sheets, different gap junction distributions, heterogeneous cell morphologies) than pure crop- and volume-dependent effects, as indicated in the results above. Based on these thoughts, we expect that the reported myocyte passive conductivities are relevant and valuable for computational modeling studies, especially if the sampling of conductivity distributions is of interest.

Table S1: Linear regression analyses of conductivities and tissue features

|  |  | Overall | | | Control | | | MI | | |
| --- | --- | --- | --- | --- | --- | --- | --- | --- | --- | --- |
| **x** | **y** | **R^2^** | **p** | **equation** | **R^2^** | **p** | **equation** | **R^2^** | **p** | **equation** |
| V_myo_ | σ_in_ | 0.34 | 1.05E-05 | y = 3.13e-04x -1.48e-02 | 0.35 | 3.83E-03 | y = 1.05e-03 x -6.15e-02 | 0.44 | 1.28E-04 | y = 2.50e-04 x -1.17e-02 |
| V_myo_ | σ_il_ | 0.69 | 1.04E-13 | y = 9.72e-03x -2.42e-01 | 0.67 | 3.48E-06 | y = 1.54e-02 x -5.67e-01 | 0.68 | 7.21E-08 | y = 7.72e-03 x -1.62e-01 |
| V_myo_ | σ_it_ | 0.01 | 5.74E-01 | y = -1.19e-04x +2.85e-02 | 0.03 | 4.81E-01 | y = 6.00e-04 x -2.04e-02 | 0 | 8.68E-01 | y = -4.31e-05 x +2.65e-02 |
| V_gj_ | σ_in_ | 0.34 | 1.01E-05 | y = 1.30e-01x -2.57e-03 | 0.32 | 5.89E-03 | y = 2.22e-01 x -9.90e-03 | 0.35 | 9.02E-04 | y = 1.67e-01 x -2.93e-03 |
| V_gj_ | σ_il_ | 0.75 | 5.35E-16 | y = 4.22e+00x +1.31e-01 | 0.4 | 1.72E-03 | y = 2.61e+00 x +2.37e-01 | 0.77 | 1.09E-09 | y = 6.10e+00 x +7.90e-02 |
| V_gj_ | σ_it_ | 0 | 9.08E-01 | y = -1.02e-02x +2.20e-02 | 0.02 | 5.19E-01 | y = -1.21e-01 x +2.65e-02 | 0.17 | 2.74E-02 | y = 4.07e-01 x +1.18e-02 |
| fibrosis | σ_in_ | 0.33 | 1.36E-05 | y = -3.01e-04x +5.56e-03 | 0.25 | 1.67E-02 | y = -9.38e-04 x +5.61e-03 | 0.45 | 1.00E-04 | y = -2.60e-04 x +5.07e-03 |
| fibrosis | σ_il_ | 0.78 | 1.74E-17 | y = -1.01e-02x +3.96e-01 | 0.42 | 1.08E-03 | y = -1.28e-02 x +4.19e-01 | 0.79 | 2.22E-10 | y = -8.56e-03 x +3.61e-01 |
| fibrosis | σ_it_ | 0 | 8.65E-01 | y = 3.50e-05x +2.13e-02 | 0.11 | 1.30E-01 | y = -1.32e-03 x +1.81e-02 | 0 | 8.00E-01 | y = -6.77e-05 x +2.49e-02 |
