# Supplementary material for: Confocal microscopy-based estimation of intracellular conductivities in myocardium for modeling of the normal and infarcted heart
Source: Comput Biol Med. Author manuscript; Available in PMC 2023 May 18. (PMC10195095; doi:10.1016/j.compbiomed.2022.105579)
Supplement: Supplemental Figures [file NIHMS1893710-supplement-Supplemental_Figures.pdf]

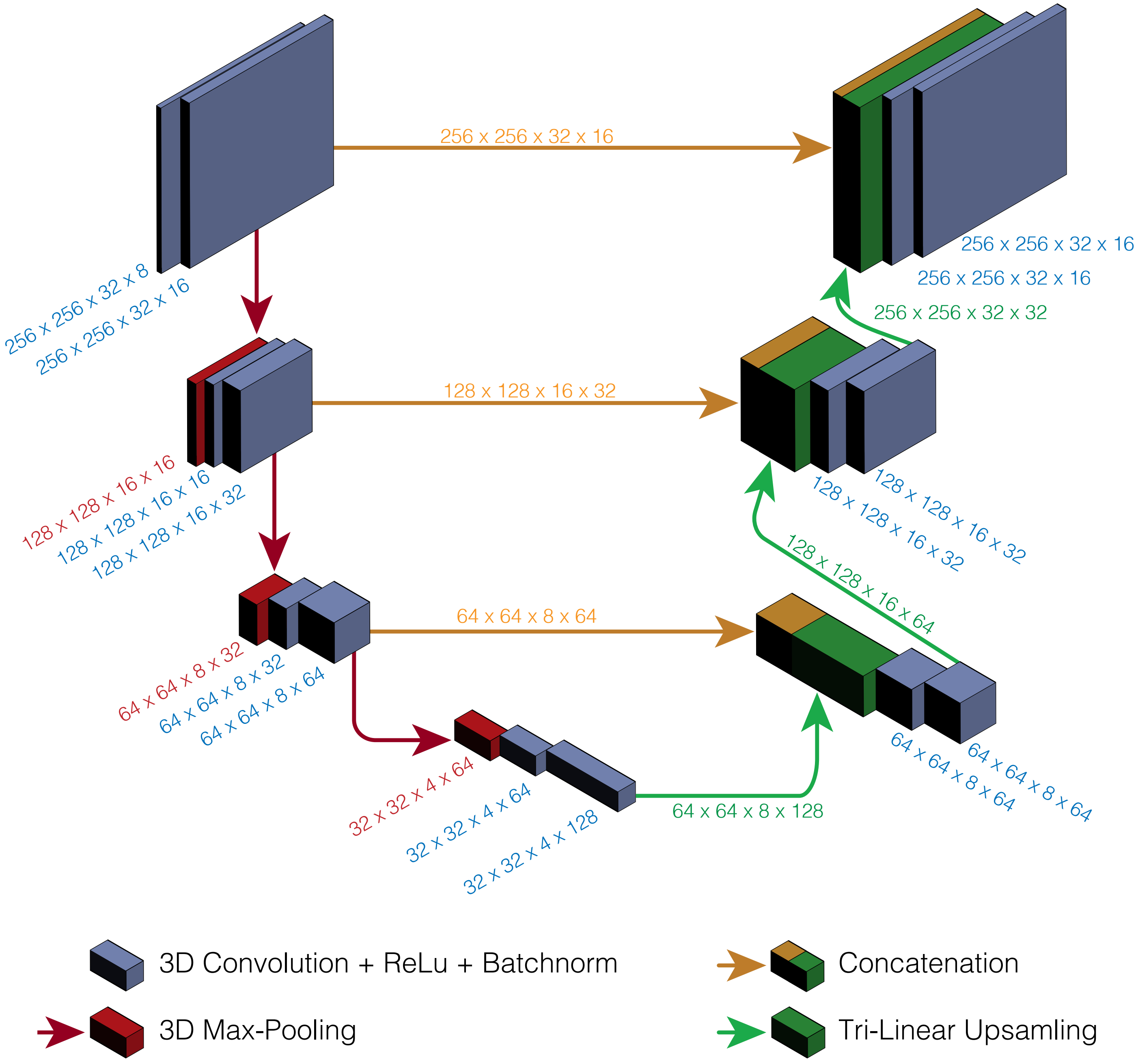

Fig. S1. 3D U-Net architecture used for the prediction of cardiomyocyte boundaries. Dimension specifications are given as width x height x depth x channels. Input to the model are image stacks from high-resolution confocal microscopy of dimension 256 x 256 x 32 x 3, where the channels correspond to signals from WGA, Cx43, and DAPI labeling.

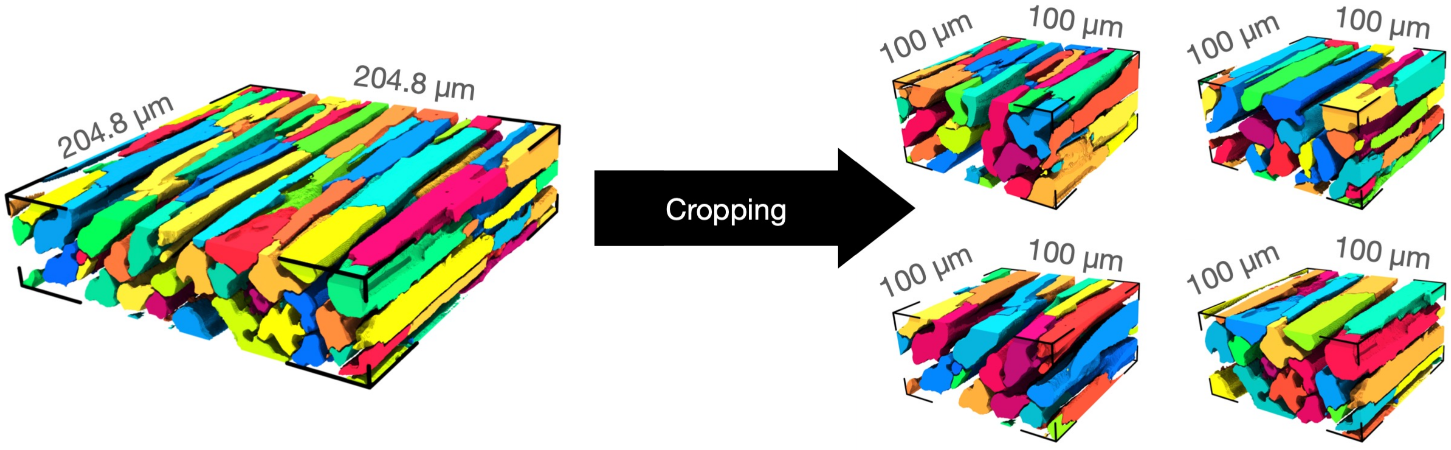

Fig. S2. Cropping strategy for the estimation of the passive myocyte conductivity tensor. Confocal microscopy image stacks and their corresponding cardiomyocyte segmentation are cropped in x- and y- direction to represent voxel sizes used in computational modeling of electrical conduction in the heart.

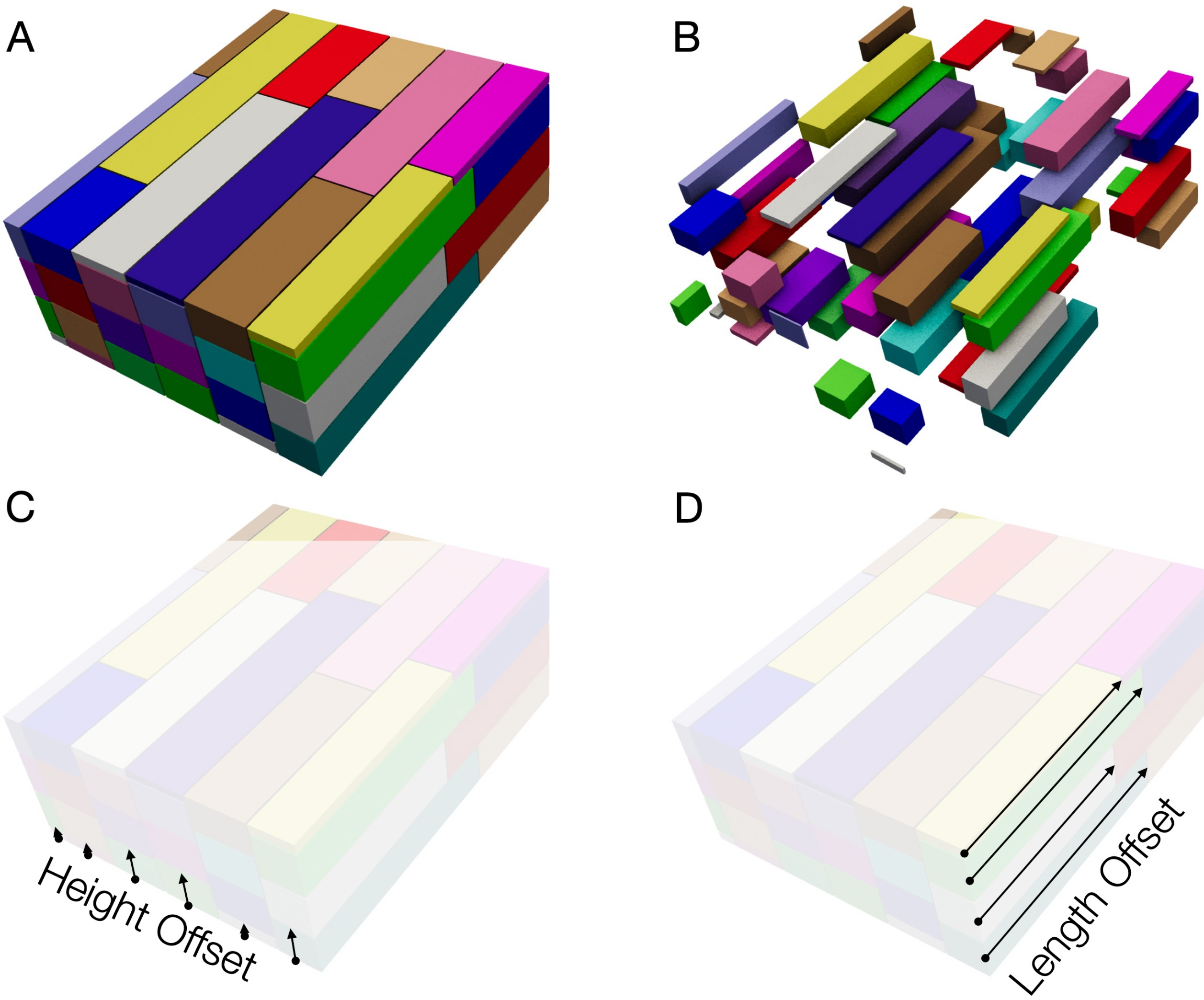

Fig. S3. Geometrical model generation for the investigation of crop-size dependence of intracellular conductivity calculation. A: Example model with a volume of 125 µm x 102.4 µm x 47.4 µm (length x width x height). **B. '**Exploded' view of **A**, visualizing the variance in cell sizes. **C:** Illustration of the random height offset per column of cells used for the generation of the geometrical model. **D:** Illustration of the random length offset per cell line of cells used for the generation of the geometrical model.

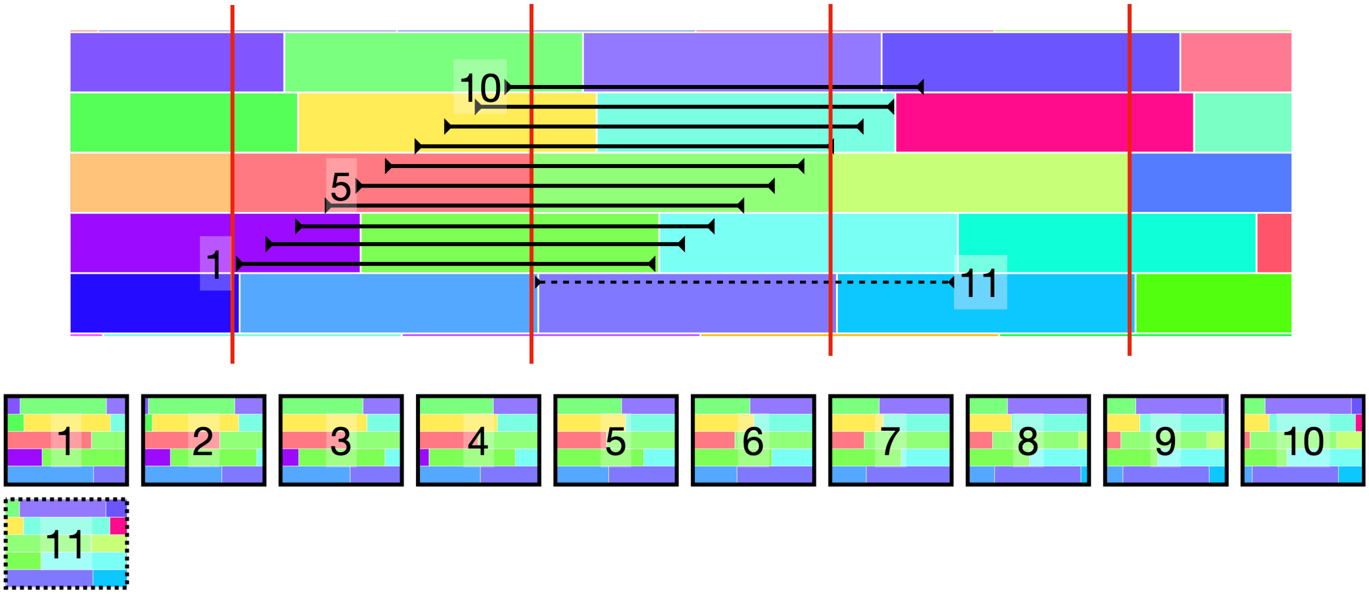

Fig. S4. Subsample strategy to investigate crop-size dependence of the passive myocyte conductivity tensor. For each realization, ten uniformly spaced crop offsets are investigated. As the model is periodic within a cardiomyocyte length (red lines), the ten crops are equally spaced within one cardiomyocyte length (0, 10, 20, 30, 40, 50, 60, 70, 80, and 90 μm).

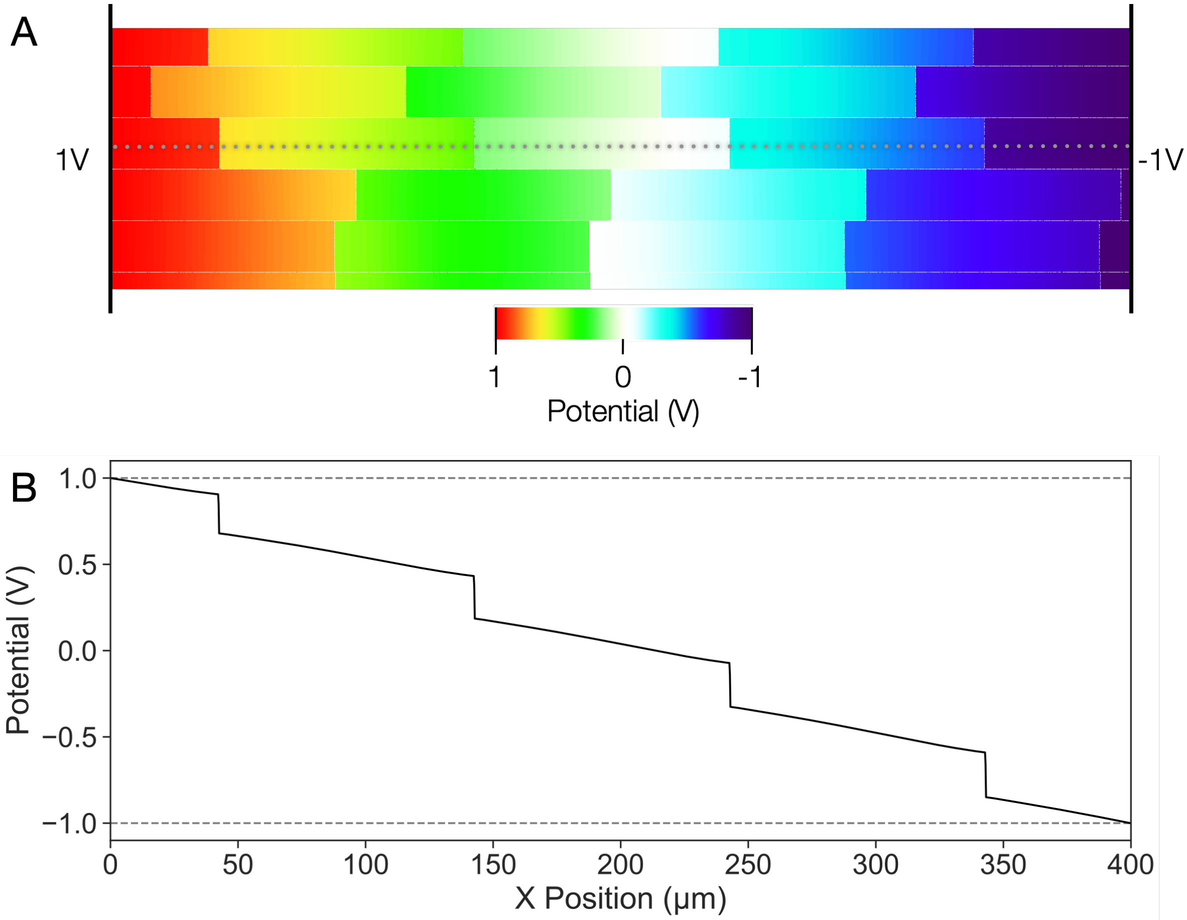

Fig. S5. Gap junction conductance normalization scheme. A: Slice through a calculated potential distribution in a normalized setup. Black bars on the left and the right represent virtual electrodes, which are implemented numerically as Dirichlet Boundary conditions. **B:** Line plot of the potential as indicated with a dotted line in A. The conductance of one cardiomyocyte is close to the total conductance formed by gap junctions between two cardiomyocytes.

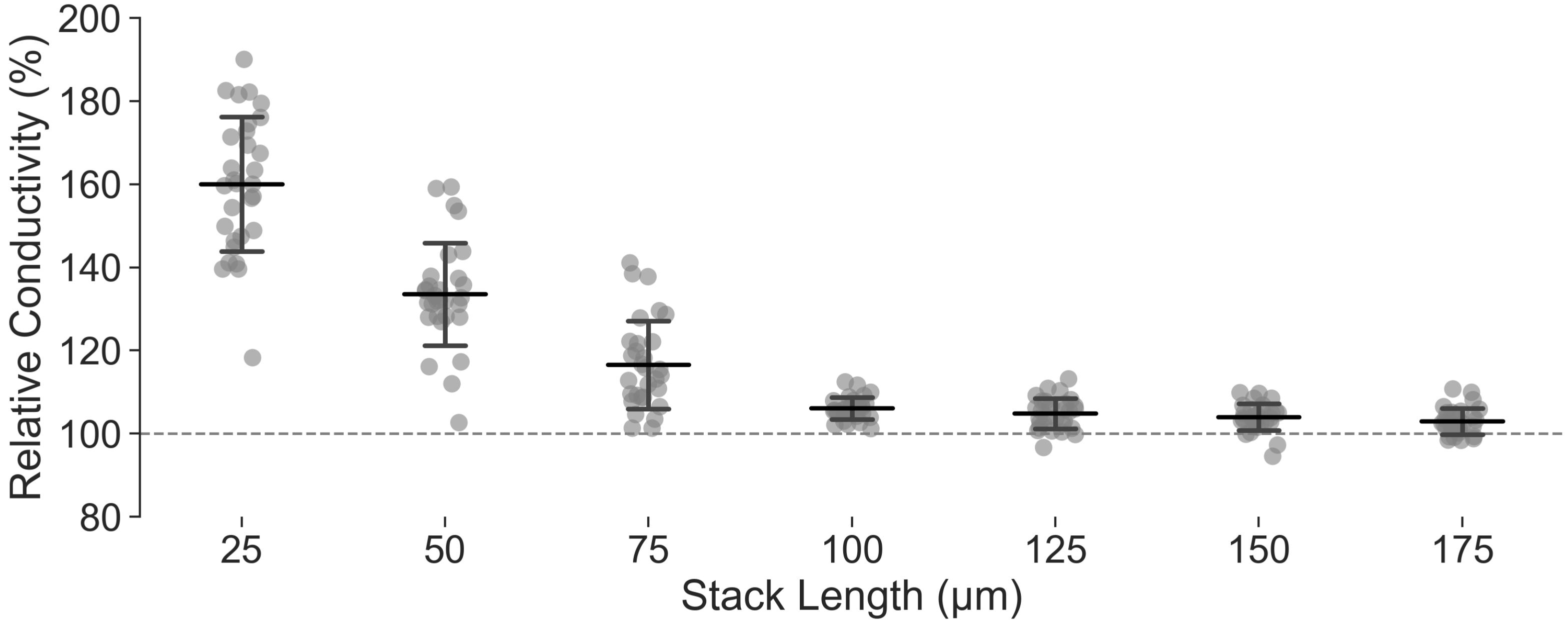

Fig. S6. Numerical analysis of stack length dependence of longitudinal myocyte conductivity. For each investigated stack length, three realizations of a myocyte-gap junction model and ten linearly spaced cropping offsets were calculated and analyzed. Displayed conductivities are normalized to the average calculated conductivities of a stack length of 400 µm. Bar and whiskers represent mean and standard deviation, respectively.

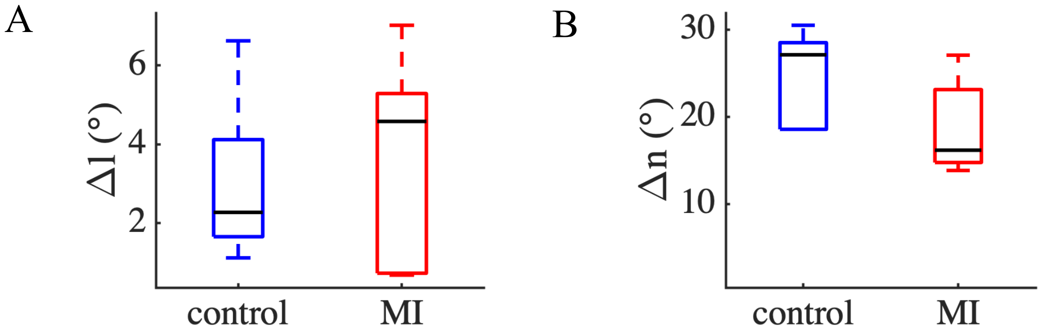

Fig. S7. **Deviation of cardiomyocyte and interlaminar cleft orientation relative to the local coordinate system set during image acquisition.** Cardiomyocyte orientation was estimated post-acquisition by PCA analysis of segmented cardiomyocytes and interlaminar clefts. We report the deviation of the orientation from the y-axis Δl and the deviation of the largest interlaminar cleft from the x-axis Δn. (A) Differences of Δl for control and MI samples were not significant. (B) Differences of Δn between control and MI samples were not significant.
